# Association of Environmental Uncertainty With Altered Decision-making and Learning Mechanisms in Youths With Obsessive-Compulsive Disorder

**DOI:** 10.1001/jamanetworkopen.2021.36195

**Published:** 2021-11-29

**Authors:** Aleya A. Marzuki, Ivan Tomić, Samantha Hiu Yan Ip, Julia Gottwald, Jonathan W. Kanen, Muzaffer Kaser, Akeem Sule, Anna Conway-Morris, Barbara J. Sahakian, Trevor W. Robbins

**Affiliations:** 1 Behavioural and Clinical Neuroscience Institute, University of Cambridge, Cambridge, United Kingdom; 2 Department of Psychology, University of Cambridge, Cambridge, United Kingdom; 3 International University Malaya–Wales, Kuala Lumpur, Malaysia; 4 Department of Public Health and Primary Care, School of Clinical Medicine, University of Cambridge, Cambridge, United Kingdom; 5 Department of Psychiatry, School of Clinical Medicine, University of Cambridge, Cambridge, United Kingdom; 6 Cambridgeshire and Peterborough NHS Foundation Trust, Cambridge, United Kingdom

## Abstract

**Importance:**

Adults with obsessive-compulsive disorder (OCD) display perseverative behavior in stable environments but exhibit vacillating choice when payoffs are uncertain. These findings may be associated with intolerance of uncertainty and compulsive behaviors; however, little is known about the mechanisms underlying learning and decision-making in youths with OCD because research into this population has been limited.

**Objective:**

To investigate cognitive mechanisms associated with decision-making in youths with OCD by using executive functioning tasks and computational modeling.

**Design, Setting, and Participants:**

In this cross-sectional study, 50 youths with OCD (patients) and 53 healthy participants (controls) completed a probabilistic reversal learning (PRL) task between January 2014 and March 2020. A separate sample of 27 patients and 46 controls completed the Wisconsin Card Sorting Task (WCST) between January 2018 and November 2020. The study took place at the University of Cambridge in the UK.

**Main Outcomes and Measures:**

Decision-making mechanisms were studied by fitting hierarchical bayesian reinforcement learning models to the 2 data sets and comparing model parameters between participant groups. Model parameters included reward and punishment learning rates (feedback sensitivity), reinforcement sensitivity and decision consistency (exploitation), and stickiness (perseveration). Associations of receipt of serotonergic medication with performance were assessed.

**Results:**

In total, 50 patients (29 female patients [58%]; median age, 16.6 years [IQR, 15.3-18.0 years]) and 53 controls (30 female participants [57%]; median age, 16.4 years [IQR, 14.8-18.0 years]) completed the PRL task. A total of 27 patients (18 female patients [67%]; median age, 16.1 years [IQR, 15.2-17.2 years]) and 46 controls (28 female participants [61%]; median age, 17.2 [IQR, 16.3-17.6 years]) completed the WCST. During the reversal phase of the PRL task, patients made fewer correct responses (mean [SD] proportion: 0.83 [0.16] for controls and 0.61 [0.31] for patients; 95% CI, −1.31 to −0.64) and switched choices more often following false-negative feedback (mean [SD] proportion: 0.09 [0.16] for controls vs 0.27 [0.34] for patients; 95% CI, 0.60-1.26) and true-positive feedback (mean [SD] proportion: 0.93 [0.17] for controls vs 0.73 [0.34] for patients; 95% CI, −2.17 to −1.31). Computational modeling revealed that patients displayed enhanced reward learning rates (mean difference [MD], 0.21; 95% highest density interval [HDI], 0.04-0.38) but decreased punishment learning rates (MD, −0.29; 95% HDI, −0.39 to −0.18), reinforcement sensitivity (MD, −4.91; 95% HDI, −9.38 to −1.12), and stickiness (MD, −0.35; 95% HDI, −0.57 to −0.11) compared with controls. There were no group differences on standard WCST measures and computational model parameters. However, patients who received serotonergic medication showed slower response times (mean [SD], 1420.49 [279.71] milliseconds for controls, 1471.42 [212.81] milliseconds for patients who were unmedicated, and 1738.25 [349.23] milliseconds for patients who were medicated) (control vs medicated MD, −320.26 [95% CI, −547.00 to −88.68]) and increased unique errors (mean [SD] proportion: 0.001 [0.004] for controls, 0.002 [0.004] for patients who were unmedicated, and 0.008 [0.01] for patients who were medicated) (control vs medicated MD, −0.007 [95% CI, −3.14 to −0.36]) on the WCST.

**Conclusions and Relevance:**

The results of this cross-sectional study indicated that youths with OCD showed atypical probabilistic reversal learning but were generally unimpaired on the deterministic WCST, although unexpected results were observed for patients receiving serotonergic medication. These findings have implications for reframing the understanding of early-onset OCD as a disorder in which decision-making is associated with uncertainty in the environment, a potential target for therapeutic treatment. These results provide continuity with findings in adults with OCD.

## Key Points

**Question:**

Is decision-making associated with environmental uncertainty in youths with obsessive-compulsive disorder (OCD)?

**Findings:**

In this cross-sectional study of 103 individuals, hierarchical reinforcement learning models fitted to 2 clinical data sets indicated that youths 12 to 19 years of age with OCD revealed atypical trial-by-trial performance on a probabilistic reversal learning 2-choice task. However, on a deterministic set-shifting task, youths with OCD did not show marked differences from healthy controls.

**Meaning:**

Obsessive-compulsive disorder in youths was associated with impaired decision-making during probabilistic tasks but not deterministic tasks, contributing to growing evidence that youths with OCD may have difficulty coping with environmental uncertainty.

## Teaser

This cross-sectional analysis investigates the association of environmental uncertainty with altered decision-making and learning mechanisms by assessing executive functioning with a probabilistic reversal learning task and the Wisconsin Card Sorting Task and by using computational modeling in youths 12 to 19 years of age with obsessive-compulsive disorder.

## Introduction

Obsessive-compulsive disorder (OCD) in adults is characterized by widespread cognitive dysfunction, particularly in domains of cognitive flexibility and response inhibition.^1,2^ Difficulties in shifting attention from ingrained thoughts and actions (inflexibility) and inhibiting inappropriate responses (response disinhibition) are thought to promote uncontrollable obsessions and urges. Curiously, evidence for these cognitive biomarkers in adolescent and child patients with OCD is sparse.^3,4^ Hence, it is now necessary to identify a cognitive biomarker that can better account for both pediatric- and adult-OCD symptomatology.

Cognition among individuals with OCD may be altered as a function of task stochasticity (ie, whether task outcomes have certain or uncertain payoffs). On deterministic set-shifting tasks, such as the Wisconsin Card Sorting Task (WCST), which involves learning from consistently reliable feedback to choose cards based on a rule (eg, color) and switching behavior when feedback changes (eg, switching from color to shape), adults with OCD typically commit more perseverative errors than healthy adults because adults with OCD inappropriately attend to previously correct rules and are slower to learn new rules.^5,6,7,8,9,10,11^ The inverse is apparent on probabilistic reversal learning (PRL) tasks. On such tasks, participants must first identify which of 2 stimuli reliably delivers positive feedback (eg, 70% of the time) and repeatedly select the more optimal stimulus on every trial to maximize rewards. When a criterion or a certain number of trials has been reached, a reversal of reward probabilities occurs, wherein the previously rewarded stimulus now becomes suboptimal (is now 30% rewarding). Recent computational studies report that adults with OCD show increased choice switching irrespective of feedback (also known as reduced stimulus stickiness) on PRL tasks.^12,13,14^ Thus, the adult OCD literature indicates a stark pattern of inflexibility when tasks are deterministic but more inconsistent choosing when tasks are probabilistic. However, there is emerging evidence that adults with OCD also display abnormal choice switching on the WCST.^15^ Researchers theorize that this aberrant choice switching is attributed to “overcomplicated exploration,” in which adults with OCD attempt to evaluate too many rules at once.^15^

It is unknown whether choice switching extends to young people with OCD. Results from this population on deterministic set-shifting tasks have been mixed.^3^ By contrast, contemporary empirical research reports that youths with OCD are prone to exhibiting poor learning, suboptimal decision-making, and increased evidence accumulation.^16,17,18^ However, studies have not fully addressed whether task stochasticity impacts decision-making.

Enhanced choice exploration during decision-making in patients with OCD could have clinical significance, in that the behavior is associated with patients doubting their decisions. These doubts may be promoting an inability to commit to a choice, even when evidence accumulated so far reveals a specific choice to be clearly more advantageous. In daily life, doubt may similarly be driving patient compulsions, such as checking locks, doors, and appliances.

Prior research probing cognition in pediatric OCD typically used frequentist statistical methods to analyze data, which may lack the sensitivity necessary to uncover more subtle behavioral anomalies. Thus, we sought to infer the mechanisms underlying learning and decision-making in juveniles with OCD by using a computational psychiatry approach. We fitted well-validated reinforcement learning models to specially acquired WCST and PRL data sets, which enabled us to investigate components of decision-making (reward and punishment sensitivity, exploration, and perseveration) that may distinguish adolescents with OCD from neurotypical adolescents. We hypothesized that youths aged 12 to 19 years with OCD would show reduced perseveration and increased exploration on the 2 tasks, which is consistent with findings in adults with OCD. Incidentally, because suitable sample sizes were available, we sought to explore the association of serotonergic medication with decision-making in patients.

## Methods

### Participants

This study is reported according to the Strengthening the Reporting of Observational Studies in Epidemiology (STROBE) guideline and was approved by the East of England–Essex Research Ethics Committee. All volunteers gave informed written consent that was obtained in a manner consistent with the East of England–Essex Research Ethics Committee requirements. Parental consent was obtained for participants younger than 16 years of age. Participants were compensated at the rate of £8 (approximately US $11) per hour.

Youths with OCD were recruited via Child and Adolescent Mental Health Services throughout the United Kingdom. Healthy control participants were recruited via advertisements in state secondary schools and notice boards located in Cambridgeshire. Patients were screened by an experienced psychiatrist to rule out comorbid psychiatric and neurological conditions. Control participants were screened to rule out neurological or psychiatric illnesses.

To qualify for the study, youths in the OCD group had to meet *Diagnostic and Statistical Manual of Mental Disorders* (Fifth Edition) (*DSM-5*) diagnostic criteria for OCD. Apart from OCD, other significant Axis I mental disorders as diagnosed according to *DSM-5* criteria were exclusion criteria. Youths with severe physical impairments affecting eyesight or motor performance were also excluded because these impairments were hypothesized to affect performance on the tasks. Participants were aged 12 to 19 years and were fluent in English.

For the WCST, 44 youths with OCD were screened, and 17 were excluded for comorbidities. For the PRL task, 104 youths with OCD were screened, and 54 were excluded for comorbidities.

### Tasks

**Figure 1.**
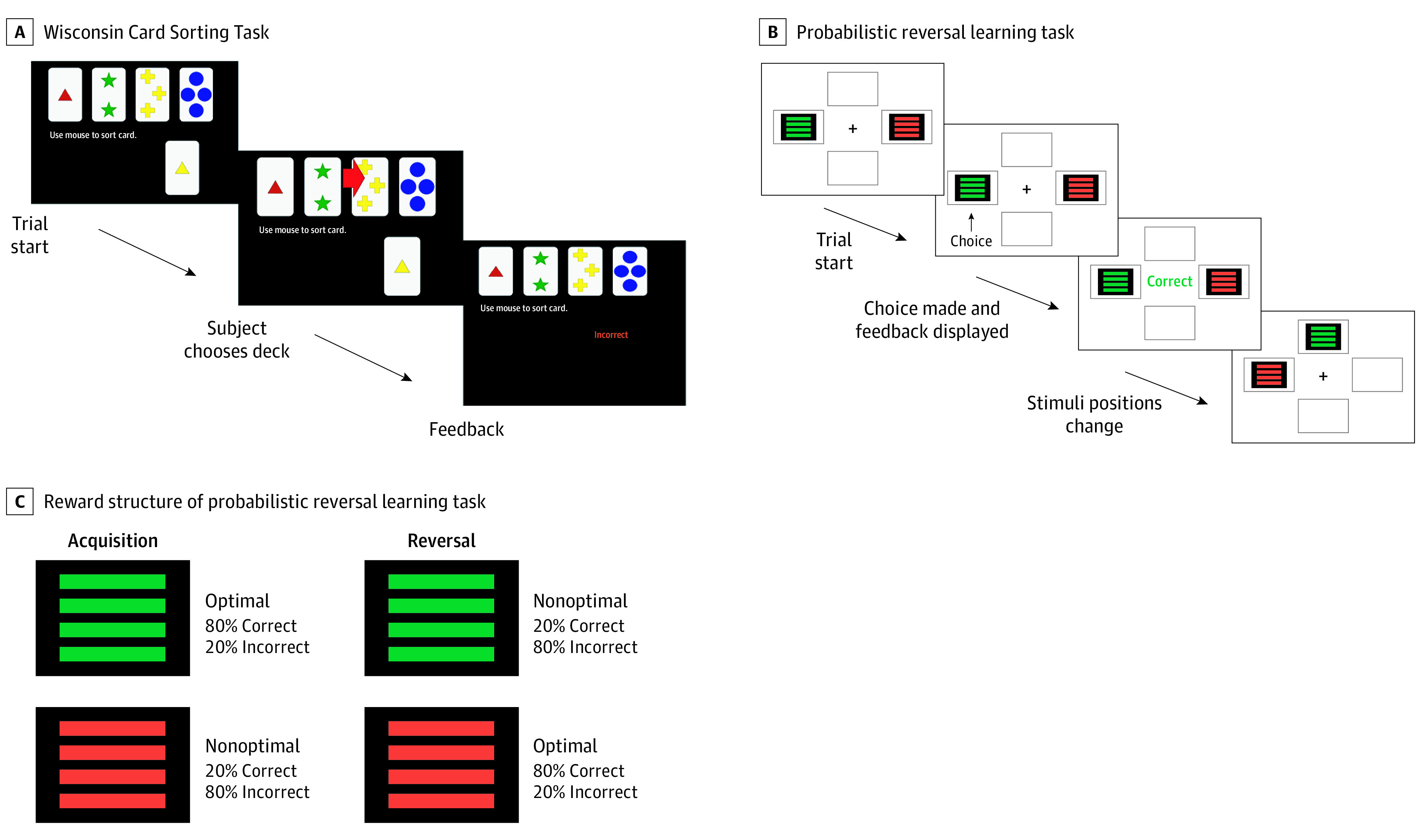
Wisconsin Card Sorting Task and Probabilistic Reversal Learning Task A, On each trial, participants sort cards appearing at the bottom of the screen into any of the 4 decks based on a rule (number, color, or shape) and use the feedback given to discern the rule. The rule changes after 10 cards have been sorted correctly consecutively. B, On every trial, participants choose between 2 colored stimuli presented on a screen. Immediately after each choice, they are given feedback. C, During the acquisition phase (first 40 trials), the stimulus chosen first provides positive feedback 80% of the time, whereas the other stimulus provides positive feedback 20% of the time. After 40 trials, the reversal phase begins with feedback contingencies associated with each stimulus reversed.

The WCST and PRL task were administered in person to participants as measures of learning and flexibility. The WCST was used to assess deterministic learning, and the PRL task probed probabilistic learning (Figure 1 and eMethods in the Supplement).

### Statistical Analysis

The outcome measures for the PRL task were the proportion of perseverative errors, that is, the number of perseverative responses made in a row immediately following reversal, the proportion of correct responses (proportion of correct choices), the proportion of switching following spurious (false) negative feedback, the proportion of staying following veridical (true) positive feedback, and the mean response times (RTs, in milliseconds). A mixed regression was used to model RT, a binomial regression was used to model p(perseverative), and mixed binomial regressions were used to model the other measures. All mixed regression models were multivariable because they included group and phase (acquisition and reversal) as independent measures.

The following outcome measures are reported for the WCST: the number of sets completed (out of 9), proportion of perseverative errors (ie, incorrectly choosing a deck based on the rule from the previous set), proportion of nonperseverative errors (errors that were not perseverative), proportion of unique errors (a deck chosen that does not match the test card on any rule), number of set maintenance failures (number of times participants chose wrongly after establishing the rule), number of trials needed to complete first set, and RT. All variables were included in multivariate linear regressions with group as an independent variable, except for proportion of perseverative errors, proportion of nonperseverative, and proportion of unique errors, which were modeled using multivariate binomial regressions.

Analyses for both tasks were repeated with *z*-scored ages, sex, and IQ included as covariates. To control for multiple comparisons, *P* values were adjusted according to the Benjamini-Hochberg procedure.^19^ Analyses for both tasks were reconducted after subdividing the OCD group into medicated and unmedicated participants. Post hoc comparisons for control, unmedicated, and medicated youths were conducted using the emmeans R package,^20^ with Bonferroni corrections applied (eMethods in the Supplement).

Data analysis was performed using RStudio, version 4.0.4 (R Foundation for Statistical Computing). Statistical significance was set at *P* < .05, and all tests were 2-tailed.

### Computational Models

To investigate mechanisms underlying decision-making, we fitted families of hierarchical bayesian reinforcement learning models to trial-by-trial task data. Models for both tasks were compared using a bridge sampling estimate of the marginal likelihood via the bridgesampling R package.^21^ The best-fitting model for the PRL data was a reinforcement learning model with 4 free parameters: reward rate, punishment rate, reinforcement sensitivity, and stickiness.^13^ The best-fitting WCST model was a sequential learning model^22,23^ with 3 free parameters: reward rate, punishment rate, and decision consistency. Reward and punishment rate parameters in both task models represented how quickly participants updated their beliefs about the values associated with choices following respective negative and positive feedback. A high punishment rate signals that a participant learns quickly from negative feedback, while a high reward rate signifies quicker learning following positive feedback. The decision consistency parameter from the WCST model and reinforcement sensitivity from the PRL model both influenced the estimated probability of choosing a specific stimulus per trial. Larger values of those parameters indicated increased exploitation (a preference for choosing the stimulus with the higher perceived value), whereas lower values indicated increased exploration (more random and less value-driven choices). Finally, stickiness (perseveration) in the PRL model described the extent to which previous choices were repeated irrespective of feedback.

We analyzed differences in parameter values between groups by first calculating group mean differences (MDs) (posterior distribution of youths in the control group minus posterior distribution of youths with OCD) per parameter. The 95% and 90% highest density intervals (HDIs)^24^ of the posterior distribution per MD were then calculated and inspected to check whether they reliably included zero (indicating no difference between groups). Full details of model formulation, model-fitting, and parameter recovery are in the eMethods and eTables 12, 13, 14, and 15 in the Supplement.

We applied Pearson correlations to assess associations between model parameters and clinical measures (eTables 16, 17, 18, 19, 20, and 21 and eFigures 5 and 6 in the Supplement). We also modeled data from 20 youths with OCD and 17 control participants who completed both the WCST and PRL task to draw more direct comparisons on behavior in both tasks (eFigure 7 in the Supplement).

## Results

A total of 50 patients with OCD (29 female patients [58%] and 21 male patients [42%]; median age, 16.6 years [IQR, 15.3-18.0 years]) and 53 control participants (30 female participants [57%] and 23 male participants [43%]; median age, 16.4 years [IQR, 14.8-18.0 years]) completed the PRL task between January 2014 and March 2020. In total, 27 youths with OCD (18 female patients [67%] and 9 male patients [33%]; median age, 16.1 years [IQR, 15.2-17.2 years]) and 46 control participants (28 female participants [61%] and 18 male participants [39%]; median age, 17.2 years [IQR, 16.3-17.6 years]) completed the WCST between January 2018 and November 2020. For the PRL sample, 30 of 50 patients were receiving selective serotonin reuptake inhibitors (SSRIs) at the time of the study. Of these patients, 20 received sertraline (mean [SD] dose, 126.25 [54.70] mg), and 10 received fluoxetine (mean [SD] dose, 35.00 [17.16] mg). For the WCST sample, 11 patients received SSRIs, and 16 patients were unmedicated; 8 patients received sertraline (mean [SD] dose, 118.75 [53.03] mg), and 3 patients received fluoxetine (mean [SD] dose, 36.67 [15.28] mg).

### Standard PRL Results

The patient and control groups were matched for age, IQ, and sex (Table 1). Analyses indicated significant interactions between group and phase on proportion of correct choices (estimated coefficient = −0.98; 95% CI, −1.31 to −0.64), proportion of switching in response to spurious (false) negative feedback (estimated coefficient = 0.93; 95% CI, 0.60-1.26), and proportion of staying in response to veridical (true) positive feedback (estimated coefficient = −1.74; 95% CI, −2.17 to −1.31) (Table 2; eFigure 1 in the Supplement). Those results indicated that, during the reversal phase, compared with controls, patients with OCD made significantly fewer correct responses (mean [SD] proportion: 0.83 [0.16] for controls vs 0.61 [0.31] for patients), switched more following spurious (false) negative feedback (mean [SD] proportion: 0.09 [0.16] for controls vs 0.27 [0.34] for patients), and stayed less following veridical (true) positive feedback (mean [SD] proportion: 0.93 [0.17] for controls vs 0.73 [0.34] for patients; 95% CI, −2.17 to −1.31). Significant results persisted when controlling for covariates (eTable 1 in the Supplement). When stratified by medication, the unmedicated and medicated groups showed reduced proportion of correct choices (control vs unmedicated patients: estimated coefficient = 1.35 [95% CI, 0.50-2.2]; control vs medicated: estimated coefficient = 1.14 [95% CI, 0.39-1.88]; unmedicated vs medicated: estimated coefficient = −0.21 [95% CI, −1.14 to 0.72]) and proportion of staying in response to veridical (true) positive feedback (control vs unmedicated patients: estimated coefficient = 2.95 [95% CI, 0.83-5.08]; control vs medicated: estimated coefficient = 2.68 [95% CI, 0.79-4.57]; unmedicated vs medicated: estimated coefficient = −0.27, [95% CI, −2.48 to 1.93]) compared with the control group during the reversal phase, but there were no significant differences between the unmedicated and medicated subgroups. Post hoc group comparisons for proportion of switching in response to spurious (false) negative feedback were not significant (eTables 2, 3, 4, and 5 and eFigure 2 in the Supplement).

**Table 1.** Summary of Demographic and Clinical Measures for the PRL Task, Control vs OCD Groups^a^

Measure	Mean (SD) value		Mean or median difference (95% CI)	*P* value
	Control (n = 53)	OCD (n = 50)		
Sex, No. (%)				
Girls	30 (56.6)	29 (58.0)	NA	.89
Boys	23 (43.4)	21 (42.0)		
Age, median (IQR), y	16.4 (14.8-18.0)	16.6 (15.3-18.0)	0.22 (−1.03 to 0.56)	.66
WASI-II (IQ) score^b^^,^^c^	109.11 (10.79)	106.57 (12.10)	2.54 (−1.95 to 7.04)	.27
BDI score^c^^,^^d^	46.81 (6.43)	59.32 (10.76)	−12.51 (−16.01 to −9.00)	<.001
BAI score^c^^,^^d^	48.04 (7.09)	62.82 (11.29)	−14.78 (−18.50 to −11.06)	<.001
OCI score, median (IQR)^c^^,^^d^	8 (4-14)	27 (18-36)	−19.00 (−23.00 to −15.00)	<.001
CY-BOCS score^b^^,^^c^	NA	23.47 (5.14)	NA	NA

Abbreviations: BAI, Beck Anxiety Inventory (*t*-scored); BDI, Beck Depression Inventory (*t*-scored); CY-BOCS, Child Yale-Brown Obsessive-Compulsive Scale; IQ, intelligence quotient; NA, not applicable; OCD, obsessive-compulsive disorder; OCI, Obsessive-Compulsive Inventory; PRL, probabilistic reversal learning; WASI-II, Weschler Abbreviated Scale of Intelligence–II.

^a^ Mean (SD) values reported for normally distributed data; median (IQR) values, for nonnormally distributed data.

^b^ Missing data from 1 participant with OCD.

^c^ Descriptions of clinical questionnaires can be found in the eMethods in the Supplement.

^d^ Significant at *P* < .05.

**Table 2.** Summary of PRL Measures and Analysis, Control vs OCD Groups

DV and IV	Estimated coefficient (95% CI)	*P* value	BH-adjusted *P* value	Test used	Mean (SD) value			
					Acquisition		Reversal	
					Control	OCD	Control	OCD
Proportion of correct choices								
Group	−0.24 (−0.75 to 0.26)	.34	.34	Binomial mixed model	0.96 (0.11)	0.94 (0.10)	0.83 (0.16)	0.61 (0.31)
Phase	−1.66 (−1.92 to −1.42)	<.001	<.001					
Group × phase	−0.98 (−1.31 to −0.64)	<.001	<.001					
Reaction time, ms								
Group	93.52 (−51.72 to 238.77)	.21	.27	Mixed linear model	1035.92 (401.98)	1129.45 (428.97)	975.41 (355.06)	1000.84 (308.71)
Phase	−60.52 (−118.32 to −2.72)	.04	.04					
Group × phase	−68.09 (−151.05 to 14.87)	.11	.11					
Proportion of switching in response to spurious (false) negative feedback								
Group	1.04 (−0.19 to 2.27)	.10	.16	Binomial mixed model	0.06 (0.14)	0.12 (0.21)	0.09 (0.16)	0.27 (0.34)
Phase	0.50 (0.25 to 0.75)	<.001	<.001					
Group × phase	0.93 (0.60 to 1.26)	<.001	<.001					
Proportion of staying in response to veridical (true) positive feedback								
Group	−1.05 (−2.20 to 0.09)	.72	.16	Binomial mixed model	0.97 (0.10)	0.95 (0.08)	0.93 (0.17)	0.73 (0.34)
Phase	−0.94 (−1.27 to −0.61)	<.001	<.001					
Group × phase	−1.74 (−2.17 to −1.31)	<.001	<.001					
Proportion of perseverative errors (only during reversal phase)								
Group	0.74 (0.57 to 0.92)	.02	.08	Binomial model	NA	NA	0.11 (0.13)	0.21 (0.27)

Abbreviations: BH, Benjamini-Hochberg correction; OCD, obsessive-compulsive disorder; DV, dependent variable; IV, independent variable; NA, not applicable; p(perseverative error); PRL, probabilistic reversal learning.

### PRL Modeling

**Figure 2.**
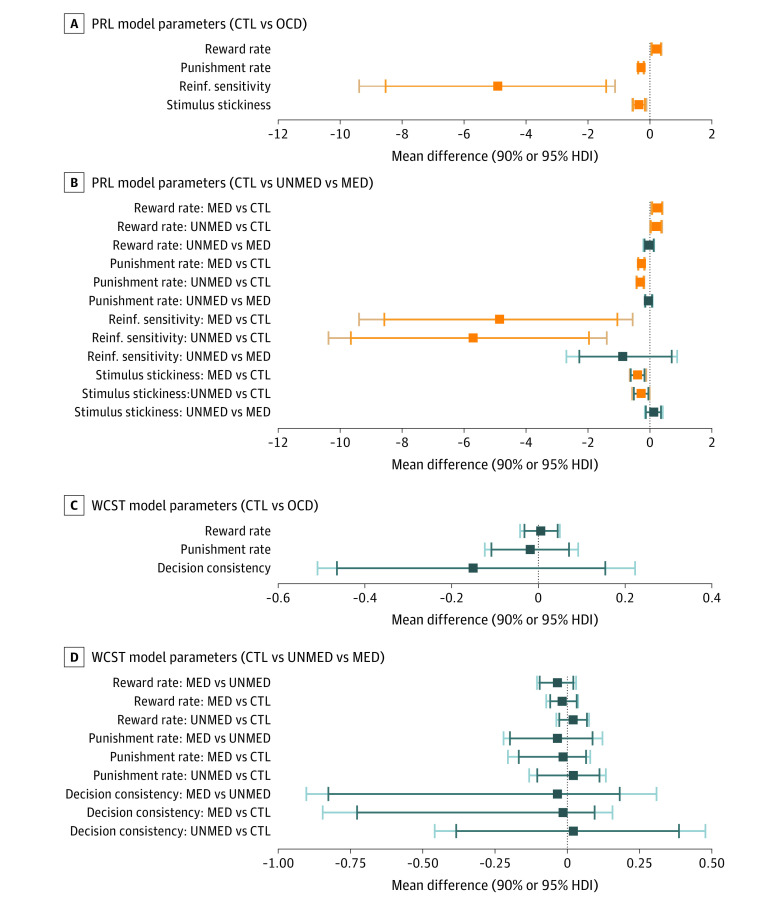
Computational Modeling Results From the Wisconsin Card Sorting Task (WCST) and the Probabilistic Reversal Learning (PRL) Task Error bars in orange indicate credible differences in posterior distributions between groups of patients with obsessive-compulsive disorder (OCD) and control (CTL) participants (not including 0) within a 95% highest density interval (HDI). A and B, Results from a winning computational model for the PRL task. A, Patients with OCD show increased reward rates but decreased punishment rates, reinforcement (Reinf.) sensitivity, and stickiness. B, Patients who were unmedicated (UNMED) and patients who were medicated (MED) show increased reward rates but decreased punishment rates, reinforcement sensitivity, and stickiness compared with CTL. There are no noticeable differences in parameter values between UNMED and MED groups. C and D, Results from a winning computational model for WCST. C, No noticeable differences in parameter values between OCD and CTL groups. D, No noticeable differences in parameter values between UNMED, MED, and CTL groups. For each parameter, the longer whiskers are 90% HDIs, and the shorter whiskers are 95% HDIs.

Compared with the control group, patients with OCD displayed increased reward rates (MD, 0.21 [95% HDI, 0.04-0.38]), lower punishment rates (MD, −0.29 [95% HDI, −0.39 to −0.18]), lower reinforcement sensitivity (MD, −4.91 [95% HDI, −9.38 to −1.12]), and lower stickiness (MD, −0.35 [95% HDI, −0.57 to −0.11]). The unmedicated and medicated subgroups also differed from the control group on all parameters, showing increased reward rates (unmedicated vs controls: MD, 0.20 [95% HDI, 0.0007-0.39]; medicated vs controls: MD, 0.24 [95% HDI, 0.04-0.41]) but decreased punishment rates (unmedicated vs controls: MD, −0.32 [95% HDI, −0.44 to −0.18]; medicated vs controls: MD, −0.28 [95% HDI, −0.39 to −0.16]), reinforcement sensitivity (unmedicated vs controls: MD, −5.73 [95% HDI, −10.37 to −1.39; medicated vs controls: MD, −4.85 [95% HDI, −9.39 to −0.59]), and stickiness (unmedicated vs controls: MD, −0.28 [95% HDI, −0.57 to −0.006; medicated vs controls: MD, −0.40 [95% HDI, −0.65 to −0.12). There were no differences between unmedicated and medicated patients across these 4 parameters (Figure 2A and B).

### Standard WCST Results

Youths in the OCD and control groups were matched for age, IQ, and sex and were equivalent on all WCST outcome measures when controlling for multiple comparisons (Table 3; eTables 6 and 7 and eFigure 3 in the Supplement). However, when subdividing the OCD group by medication status, there was a significant association of group with RT (estimated coefficient = 138.29; 95% CI, 50.40-226.18) and proportion of unique errors (estimated coefficient = 0.89; 95% CI, 0.29-1.51). Post hoc comparisons indicated that patients who received medication (mean [SD], 1738.25 [349.23] milliseconds) displayed significantly slower mean (SD) response times than control participants (1420.49 [279.71] milliseconds) but not slower than patients who received no medication (1471.42 [212.81] milliseconds) (control vs medicated: MD, −320.26 [95% CI, −547.00 to −88.68]; medicated vs unmedicated: MD, −269.33 [95% CI, −534 to 0.50]; unmedicated vs control: MD, −50.93 [95% CI, −249.00 to 147.17]). Moreover, patients who received medication (mean [SD] proportion, 0.008 [0.01]) showed increased unique errors compared with control participants (mean [SD] proportion, 0.001 [0.004]), but there were no significant differences between patients who did and who did not receive medication (mean [SD] proportion, 0.002 [0.004]), nor between control participants and patients who received no medication (control vs medicated: MD, −0.007 [95% CI, −3.14 to −0.36]; medicated vs unmedicated: MD, −0.006 [95% CI, −2.77 to 0.45]; unmedicated vs control: MD, −0.001 [95% CI, −2.29 to 1.14]). Significant group differences were maintained when controlling for covariates (eTables 8, 9, 10, and 11 and eFigure 4 in the Supplement).

**Table 3.** Summary of Clinical, Demographic, and Wisconsin Card Sorting Task Outcome Scores (Control vs OCD)^a^

Outcome	Mean (SD)		Mean or median difference (95% CI)	*P* value^b^
	Control (n = 46)	OCD (n = 27)		
Sex, No. (%)				
Girls	28 (60.9)	18 (66.7)	NA	.62
Boys	18 (39.1)	9 (33.3)		
Age, median (IQR), y	17.2 (16.3-17.6)	16.1 (15.2-17.2)	1.09 (−0.08 to 1.42)	.07
WASI-II (IQ) score^c^	107.61 (11.62)	107.12 (13.02)	−0.49 (−5.69 to 6.68)	.87
BDI score^d^	46.46 (5.27)	59.04 (9.55)	−12.58 (−16.63 to −8.53)	<.001
BAI score, median (IQR)^d^	46 (44-50)	64 (59-69)	−18 (−22 to −14)	<.001
OCI score, median (IQR)^d^	6.5 (4.0-11.0)	28.0 (23.5-36.5)	−21.5 (−26.0 to −18.0)	<.001
CY-BOCS score^c^	NA	23.62 (4.84)	NA	NA
No. of sets completed	7.96 (1.48)	7.52 (2.03)	−0.44 (−1.26 to 0.38)	BH-adjusted *P* = .34 (−0.44)
Proportion of perseverative errors	0.12 (0.03)	0.13 (0.07)	0.01 (−0.14 to 0.26)	BH-adjusted *P* = .54 (0.06)
Proportion of nonperseverative errors	0.06 (0.04)	0.08 (0.06)	0.02 (−0.07 to 0.66)	BH-adjusted *P* = .21 (0.30)
Reaction time, ms	1420.49 (279.72)	1580.13 (301.48)	159.64 (20.47 to 298.80)	BH-adjusted *P* = .09 (159.64)
Failure to maintain set	0.94 (1.06)	1.48 (1.50)	0.54 (−0.05 to 1.15)	BH-adjusted *P* = .17 (0.55)
No. of trials needed to complete first set	14.80 (9.58)	18.15 (15.49)	3.35 (−2.50 to 9.19)	BH-adjusted *P* = .34 (3.34)
Proportion of unique errors	0.001 (0.005)	0.005 (0.008)	0.004 (0.16 to 2.44)	BH-adjusted *P* = .12 (1.23)

Abbreviations: BAI, Beck Anxiety Inventory (*t*-scored); BDI, Beck Depression Inventory (*t*-scored); BH, Benjamini-Hochberg correction; CY-BOCS, Child Yale-Brown Obsessive-Compulsive Scale; IQ, intelligence quotient; NA, not applicable; OCD, obsessive-compulsive disorder; OCI, Obsessive-Compulsive Inventory; WASI-II, Weschler Abbreviated Scale of Intelligence–II.

^a^ Mean (SD) values are reported for normally distributed data; median (IQR) values are reported for nonnormally distributed data.

^b^ The BH-corrected *P* values (and estimated regression coefficients) are reported from regression analyses of Wisconsin Card Sorting Task outcome measures.

^c^ Missing data from 1 participant with OCD.

^d^ Significant at *P* < .05.

### WCST Modeling

There were no differences between youths in the OCD and control groups on reward rate (MD, 0.005 [95% HDI, −0.04 to 0.05], punishment rate (MD, −0.02 [95% HDI, −0.12 to 0.09], and decision consistency (MD, −0.15 [95% HDI, −0.51 to 0.22] parameters, or among comparisons of youths in the unmedicated, medicated, and control groups on these 3 parameters (Figure 2C and D).

## Discussion

This is the first study, to our knowledge, to fractionate cognitive processes contributing to behavior on set-shifting and decision-making paradigms in large samples of youths with OCD. We sought to understand whether youths 12 to 19 years of age with OCD, similar to adult patients with OCD, show altered choices when outcomes are either deterministic or probabilistic. On the PRL task, patients with OCD made significantly more incorrect responses and showed more switching following spurious (false) negative feedback and veridical (true) positive feedback in the reversal phase. Our computational modeling results indicated that, compared with healthy participants, the young patients with OCD, regardless of medication status, had increased reward rates and choice exploration (low reinforcement sensitivity) alongside lower punishment rates and stickiness. Youths with OCD had intact WCST performance and did not differ from control participants on any model parameters investigated. However, patients who received medication exhibited slower response times and increased unique errors compared with healthy controls.

The increased exploration displayed by patients with OCD indicates fewer value-driven decisions (ie, a greater tendency to choose the less optimal stimulus), whereas lower stickiness signifies increased switching between choices. Recent findings denote choice volatility on probabilistic reversal tasks to be a key feature of OCD.^12,25^ The present study contributes to an emerging literature showing that choice volatility also exists in youths with OCD during probabilistic tasks. The increased exploration and lower stickiness observed may have resulted from increased uncertainty regarding choices because patients with OCD commonly report more subjective uncertainty than healthy people do.^26^ By contrast, the deterministic feedback on the WCST triggers less uncertainty. Indeed, increased information-seeking behavior and exploration have been detected in adults and children with OCD, particularly on tasks in which uncertainty is enhanced or when payoffs are probabilistic.^17,27,28,29^ Anxiety is commonly evoked by uncertainty,^30^ and it is plausible that the stress experienced by patients led to disorganized patterns of responding. Response volatility may also result from attentional lapses, which may affect learning and is consistent with research reporting learning impairments in juvenile patients with OCD.^16^

Elevated reward and reduced punishment rates in our sample of youths with OCD is a novel finding because prior studies typically report increased sensitivity to negative feedback in adults with OCD.^31,32,33^ By contrast, decreased frontostriatal brain activation following positive and negative feedback has been detected in children with OCD compared with controls,^18^ suggesting blunted feedback processing in young patients. Emerging research reveals that healthy younger people are significantly more punishment averse than healthy adults.^34^ In contrast to healthy youths, those with OCD may not be particularly sensitive to either kind of feedback given that they favor exploratory over value-driven decision-making. This account is compatible with the clinical presentation of OCD in which patients’ thoughts and rituals are out of proportion to the information available in the external environment. A competing explanation is that anxiety experienced by patients enhanced learning about positive outcomes but decreased avoidance of punishment, a phenomenon previously shown by inducing stress in healthy people and possibly associated with modulation of the dopaminergic reward system.^35,36^

Because youths with OCD in this study did not differ from healthy controls on the proportion of perseverative errors made on the WCST, we conclude an absence of a cognitive flexibility deficit in this sample, consistent with previous findings.^3^ This strengthens the notion that youths with OCD differ cognitively from adult patients because the latter tend to show deficits on this task.^15,37,38^ Hence, it is plausible that cognitive flexibility becomes increasingly affected as a function of the duration of the disorder. There may also be phenotypic differences between child and adult subtypes of OCD given that cognitive inflexibility is considered a genetic marker for disorder risk.^2,39^ Future longitudinal research would be appropriate for probing competing explanations for distinctions between subtypes.

Patients receiving SSRIs in the present study displayed enhanced unique errors and slower response times. Unique errors are usually made by very young children,^40^ who have difficulty attending to and recognizing different task rules. Hence, the patients in our study who received SSRIs may have attentional and rule-learning impairments, corroborated by previous findings in adolescents with OCD.^16^ The slow responding of patients receiving medication is reminiscent of research identifying slower goal-directed planning and information-seeking in youths with OCD.^17,41,42,43^ Slowness may arise from either meticulousness or intrusive thoughts of patients with OCD.^44^

One other study to date, to our knowledge, has reported adverse effects of SSRIs on WCST performance in pediatric patients with OCD,^45^ whereas other studies have reported either null or positive effects of medication.^46,47^ An explanation for our findings is that the group receiving medications may have had a more severe form of the disorder that necessitated treatment with psychotropic medication. Alternatively, cognitive and memory deficits may occur during early stages of SSRI treatment,^48^ potentially associated with anxiolytic effects triggered initially by medication that improve over time.

### Limitations

A limitation of the present study is the relatively small sample of youths with OCD completing the WCST, particularly when grouped by medication status. Although the models fit to data were well validated for the specific tasks used, we did not consider alternative models, such as counterfactual,^34^ bayesian inference,^49,50^ and drift-diffusion models,^51,52^ that may offer alternative insight into underlying behavior.

To rule out the possibility that group differences were associated with non-OCD symptoms, we excluded patients with comorbid conditions. However, noncomorbid OCD is not wholly representative of pediatric patients with OCD, who commonly present with at least 1 comorbid disorder.^53^ Future research should recruit a naturalistic sample of patients or classify decision-making based on comorbidity profiles (eg, OCD with anxiety vs OCD with depression). Finally, future work should administer cognitive tasks using stimuli that provoke OCD symptoms because results from such tasks may have clearer clinical implications.

## Conclusions

The findings of this cross-sectional study suggested that decision-making was altered in youths with OCD in a task with probabilistic feedback but was relatively unaffected on the WCST, a deterministic test of flexibility. Our computational modeling findings suggested that environmental uncertainty promoted altered feedback learning and enchanced choice exploration in youths with OCD, possibly by triggering doubt or indecisiveness in these young patients. Moreover, choice vacillation has now been detected in both adults and adolescents with OCD, suggesting that it is a stable feature of the disorder.

## Notes

Supplement. **eMethods.** Supplemental Methods **eTable 1.** Standard Mixed Regression Results for PRL Covarying for Age, Gender, and IQ **eFigure 1.** Group Comparisons (CTL vs OCD) Across All PRL Measures **eTable 2.** Summary Statistics for Demographic and Clinical Measures (CTL vs MED- vs MED+) **eTable 3.** Summary Statistics for PRL Task Measures (CTL vs MED- vs MED+) **eTable 4.** Standard Regression Results for PRL Without Covariates (CTL vs MED- vs MED+) **eTable 5.** Standard Regression Results for PRL Covarying for Age, Gender, and IQ (CTL vs MED- vs MED+) **eFigure 2.** Group Comparisons (CTL vs MED- vs MED+) Across All PRL Measures **eTable 6.** Standard Regression Results for WCST Without Covariates (CTL vs OCD) **eTable 7.** Standard Regression Results for WCST Covarying for Age, Gender, and IQ (CTL vs OCD) **eFigure 3.** Group Comparisons (CTL vs OCD) Across All WCST Measures **eTable 8.** Summary Statistics for Demographic and Clinical Measures (CTL vs MED- vs MED+) **eTable 9.** Scores per Outcome Measure per Group (CTL vs MED- vs MED+) **eTable 10.** Standard Regression Results for WCST Without Covariates (CTL vs MED- vs MED+) **eTable 11.** Standard Regression Results for WCST Covarying for Age, Gender, and IQ (CTL vs MED- vs MED+) **eFigure 4.** Group Comparisons (CTL vs MED- vs MED+) per WCST Measure **eTable 12.** Model Comparison Using Bridgesampling (PRL) **eTable 13.** Model Comparison Using Bridgesampling (WCST) **eTable 14.** Parameter Recovery Analysis With Simulated Data Generated by Best-Fit Computational Model (CTL vs OCD) **eTable 15.** Parameter Recovery Analysis With Simulated Data Generated by Best-Fit Computational Model (CTL vs MED- vs MED+) **eTable 16.** Correlations Between Demographic/Clinical Measures and Model Parameters (PRL)—All Subjects **eTable 17.** Correlations Between Demographic/Clinical Measures and Model Parameters (PRL)—CTL Only **eTable 18.** Correlations Between Demographic/Clinical Measures and Model Parameters (PRL)—OCD Only **eFigure 5.** Plots of Correlations Between Demographic/Clinical Measures and Model Parameters (PRL) **eTable 19.** Correlations Between Demographic/Clinical Measures and Model Parameters (WCST)—All Subjects **eTable 20.** Correlations Between Demographic/Clinical Measures and Model Parameters (WCST)—CTL Only **eTable 21.** Correlations Between Demographic/Clinical Measures and Model Parameters (WCST)—OCD Only **eFigure 6.** Plots of Correlations Between Demographic/Clinical Measures and Model Parameters (WCST) **eFigure 7.** Modelling Results From 20 OCD and 17 CTL
eReferences.
