# Error in Byline

**DOI:** 10.1001/jamanetworkopen.2021.45564

**Published:** 2021-12-29

In the Original Investigation titled “Association of Environmental Uncertainty With Altered Decision-making and Learning Mechanisms in Youths With Obsessive-Compulsive Disorder,”^1^ published November 29, 2021, the seventh author should have only 1 degree (Akeem Sule, MD), not 2 degrees (Akeem Sule, MD, PhD). This article has been corrected.^1^

## References

[1] Marzuki AA, Tomić I, Ip SHY, . Association of environmental uncertainty with altered decision-making and learning mechanisms in youths with obsessive-compulsive disorder. JAMA Netw Open. 2021;4(11):e2136195. doi:10.1001/jamanetworkopen.2021.36195 34842925 PMC8630570

