# Supplementary material for: Association of Environmental Uncertainty With Altered Decision-making and Learning Mechanisms in Youths With Obsessive-Compulsive Disorder
Source: JAMA Netw Open. 2021 Nov 29;4(11):e2136195. doi: 10.1001/jamanetworkopen.2021.36195 (PMC8630570; doi:10.1001/jamanetworkopen.2021.36195)
Supplement: Supplement. — eMethods. Supplemental Methods eTable 1. Standard Mixed Regression Results for PRL Covarying for Age, Gender, and IQ eFigure 1. Group Comparisons (CTL vs OCD) Across All PRL Measures eTable 2. Summary Statistics for Demographic and Clinical Measures (CTL vs MED- vs MED+) eTable 3. Summary Statistics for PRL Task Measures (CTL vs MED- vs MED+) eTable 4. Standard Regression Results for PRL Without Covariates (CTL vs MED- vs MED+) eTable 5. Standard Regression Results for PRL Covarying for Age, Gender, and IQ (CTL vs MED- vs MED+) eFigure 2. Group Comparisons (CTL vs MED- vs MED+) Across All PRL Measures eTable 6. Standard Regression Results for WCST Without Covariates (CTL vs OCD) eTable 7. Standard Regression Results for WCST Covarying for Age, Gender, and IQ (CTL vs OCD) eFigure 3. Group Comparisons (CTL vs OCD) Across All WCST Measures eTable 8. Summary Statistics for Demographic and Clinical Measures (CTL vs MED- vs MED+) eTable 9. Scores per Outcome Measure per Group (CTL vs MED- vs MED+) eTable 10. Standard Regression Results for WCST Without Covariates (CTL vs MED- vs MED+) eTable 11. Standard Regression Results for WCST Covarying for Age, Gender, and IQ (CTL vs MED- vs MED+) eFigure 4. Group Comparisons (CTL vs MED- vs MED+) per WCST Measure eTable 12. Model Comparison Using Bridgesampling (PRL) eTable 13. Model Comparison Using Bridgesampling (WCST) eTable 14. Parameter Recovery Analysis With Simulated Data Generated by Best-Fit Computational Model (CTL vs OCD) eTable 15. Parameter Recovery Analysis With Simulated Data Generated by Best-Fit Computational Model (CTL vs MED- vs MED+) eTable 16. Correlations Between Demographic/Clinical Measures and Model Parameters (PRL)—All Subjects eTable 17. Correlations Between Demographic/Clinical Measures and Model Parameters (PRL)—CTL Only eTable 18. Correlations Between Demographic/Clinical Measures and Model Parameters (PRL)—OCD Only eFigure 5. Plots of Correlations Between Demographic/Clinical Measures and Model Param [file jamanetwopen-e2136195-s001.pdf]

# Supplementary Online Content

Marzuki AA, Tomić I, Ip SHY, et al. Association of environmental uncertainty with altered decision-making and learning mechanisms in youths with obsessive-compulsive disorder. *JAMA Netw Open*. 2021;4(11):e2136195. doi:10.1001/jamanetworkopen.2021.36195

**eMethods.** Supplemental Methods

**eTable 1.** Standard Mixed Regression Results for PRL Covarying for Age, Gender, and IQ

**eFigure 1.** Group Comparisons (CTL vs OCD) Across All PRL Measures

**eTable 2.** Summary Statistics for Demographic and Clinical Measures (CTL vs MED- vs MED+)

**eTable 3.** Summary Statistics for PRL Task Measures (CTL vs MED- vs MED+)

**eTable 4.** Standard Regression Results for PRL Without Covariates (CTL vs MED- vs MED+)

**eTable 5.** Standard Regression Results for PRL Covarying for Age, Gender, and IQ (CTL vs MED- vs MED+)

**eFigure 2.** Group Comparisons (CTL vs MED- vs MED+) Across All PRL Measures

**eTable 6.** Standard Regression Results for WCST Without Covariates (CTL vs OCD)

**eTable 7.** Standard Regression Results for WCST Covarying for Age, Gender, and IQ (CTL vs OCD)

**eFigure 3.** Group Comparisons (CTL vs OCD) Across All WCST Measures

**eTable 8.** Summary Statistics for Demographic and Clinical Measures (CTL vs MED- vs MED+)

**eTable 9.** Scores per Outcome Measure per Group (CTL vs MED- vs MED+)

**eTable 10.** Standard Regression Results for WCST Without Covariates (CTL vs MED- vs MED+)

**eTable 11.** Standard Regression Results for WCST Covarying for Age, Gender, and IQ (CTL vs MED- vs MED+)

**eFigure 4.** Group Comparisons (CTL vs MED- vs MED+) per WCST Measure

**eTable 12.** Model Comparison Using Bridgesampling (PRL)

© 2021 Marzuki AA et al. *JAMA Network Open*.

**eTable 13.** Model Comparison Using Bridgesampling (WCST)

**eTable 14.** Parameter Recovery Analysis With Simulated Data Generated by Best-Fit Computational Model (CTL vs OCD)

**eTable 15.** Parameter Recovery Analysis With Simulated Data Generated by Best-Fit Computational Model (CTL vs MED- vs MED+)

**eTable 16.** Correlations Between Demographic/Clinical Measures and Model Parameters (PRL)—All Subjects

**eTable 17.** Correlations Between Demographic/Clinical Measures and Model Parameters (PRL)—CTL Only

**eTable 18.** Correlations Between Demographic/Clinical Measures and Model Parameters (PRL)—OCD Only

**eFigure 5.** Plots of Correlations Between Demographic/Clinical Measures and Model Parameters (PRL)

**eTable 19.** Correlations Between Demographic/Clinical Measures and Model Parameters (WCST)—All Subjects

**eTable 20.** Correlations Between Demographic/Clinical Measures and Model Parameters (WCST)—CTL Only

**eTable 21.** Correlations Between Demographic/Clinical Measures and Model Parameters (WCST)—OCD Only

**eFigure 6.** Plots of Correlations Between Demographic/Clinical Measures and Model Parameters (WCST)

**eFigure 7.** Modelling Results From 20 OCD and 17 CTL

**eReferences.**

This supplementary material has been provided by the authors to give readers additional information about their work.

© 2021 Marzuki AA et al. *JAMA Network Open*.

## eMethods. Supplemental Methods

### Clinical and Cognitive Questionnaires

The Mini International Neuropsychiatric Interview (MINI for participants over 18, MINI-KID for participants under 18[1,2]) was used by clinicians to screen patients and controls for this study. Self-reported obsessive-compulsive traits were assessed using the Obsessive-Compulsive Inventory-Revised (OCI-R)[3]. OCD symptom severity was also assessed with the Children's Yale-Brown Obsessive Compulsive Scale (CY-BOCS)[4]. The CY-BOCS was only administered to participants with OCD. To obtain a measure of anxiety and depression, all participants completed the Beck Anxiety Inventory for Youth and the Beck Depression Inventory for Youth[5]. IQ measures were obtained using the Wechsler's Abbreviated Scale of Intelligence, Second Edition (WASI-II)[6]. The Full-Scale IQ-2 subtests (FSIQ-2) from the WASI-II were used comprising the Vocabulary and Matrix Reasoning tests.

IQ and CY-BOCS data from 1 OCD/MED- (unmedicated) participant was missing from this study.

### PRL

The paradigm used here is identical to the task originally used by Murphy et al. (2008)[7]. Participants were shown two stimuli, composed of four red and four green lines, on a laptop screen. They were instructed to choose either stimulus on every trial by touching it with their finger on the screen. The following written instructions were provided before the task began:

"On the screen there are two patterns, one red and one green. On each go, you must choose one of these colours and the computer will tell you whether your choice is correct or wrong. Each colour will sometimes be correct and sometimes be wrong, but one of the colours will tend to be correct more often than the other. What you have to do is find out which colour is usually correct, choose that colour every time, and stick with it even if it is occasionally wrong. At some point the rule may change so that the other colour is usually correct, in which case you should choose that one every time."

The task consisted of 80 trials in total and was split into Acquisition and Reversal phases, each consisting of 40 trials. The Acquisition phase required participants to discriminate between the optimal and non-optimal stimuli. The optimal stimulus was programmed to provide positive feedback ('Correct') on 80% of trials and negative feedback ('Incorrect') on 20% of trials. The non-optimal stimulus was programmed to provide negative feedback on 80% of trials and positive feedback on 20% of trials. The stimulus chosen by participants on the first trial was assigned as the optimal stimulus for the rest of the Acquisition phase. Subsequently during the Reversal phase, the positive to negative feedback ratio associated with each stimulus was reversed. In other words, the stimulus that was previously optimal became non-optimal and vice versa.

On correct trials, the word 'Correct' would be displayed in green alongside a consonant tone, whereas on incorrect trials, the word 'Incorrect' would be displayed in red alongside a dissonant tone. There was no time limit for responding in each trial. The task duration was approximately 7 minutes in total.

© 2021 Marzuki AA et al. *JAMA Network Open*.

### WCST

The WCST used in this study was run on a laptop via the Psychology Experiment Building Language programme[8]. The WCST contains up to 128 trials. Participants were shown 4 decks with a different combination of colours, numbers, and shapes. They were instructed to sort cards appearing at the bottom of the screen, using a computer mouse, according to one of three rules at a time, either number, colour or shape. The rule must be discovered using trial and error via visual feedback received after each card is sorted. Cards were sorted by clicking on the chosen deck using the laptop mousepad. If a card is sorted correctly, the feedback shown would be 'Correct'. If the card is sorted incorrectly, the feedback shown would be 'Incorrect'. There was no time limit for a card to be sorted on each trial, but participants were told to answer as quickly and as accurately as possible.

After 10 cards have been successfully sorted consecutively, one set is completed and the sorting rule changes. The process continues until the participant either sorts all 128 cards or they complete 9 sets. The task took approximately 10 minutes to complete.

### Standard Statistical Analysis for PRL

p(switching following SNF) and p(staying following VPF) were calculated by counting the number of times each participant switched following SNF and stayed following VPF and dividing these values by the number of times participants had the opportunity to carry out these switching and staying behaviours. p(correct) was also calculated by dividing the number of correct responses per subject by the number of trials in Reversal and Acquisition phases. Multiple multivariate mixed-binomial regressions were conducted using the glmer function from the lme4 R package[9] to assess the effects of Group (OCD vs CTL) and Phase (Acquisition vs Reversal) on the p(correct), p(switching following SNF), and p(staying following VPF) measures. A univariate binomial regression was used to analyse the effects of Group on p(perseverative errors) and did not include Phase as an independent variable as we could only study perseverative responses in the Reversal phase. RT was analysed using a multivariate mixed-linear model using the 'lmer' function. We verified that the residuals of reaction times followed a normal distribution, using a Shapiro-Wilkes test, and displayed homoscedascity, using a Breusch-Pagan test.

### Standard Statistical Analysis for WCST

The 7 outcome measures we explored (as reported in the main text) are ones that are commonly analysed in studies utilising the WCST[10–15].

Homoscedascity of residuals obtained from each regression model were assessed using the Breusch-Pagan test. When the assumption of homoscedascity of residuals was violated, a sandwich variance estimate function from the 'sandwich' R package[16] was applied to the regression model(s). This enabled the extraction of standard errors that were robust to non-constant variance. P-values and 95% confidence intervals were calculated using these new standard errors.

*Analysis for both tasks*

For both tasks, Wald 95% confidence intervals for all regression models were obtained using the 'confint' function in R.

Also for both tasks, overdispersion in the binomial models was assessed using the 'check_overdispersion' function in the 'performance' R package[17]. Quasibinomial models were implemented instead of binomial models when models showed overdispersion.

© 2021 Marzuki AA et al. *JAMA Network Open*.

**Computational Models for PRL**

To better understand processes underlying learning and decision-making on the PRL, a family of model-free reinforcement learning (RL) models were fitted to data. Modelling first involves formulating a mathematical function equipped with different parameters of interest to analyse trial-by-trial data. Values of these parameters are then estimated when the model is fit to participants' data, enabling quantification and classification of behaviour.

RL models are a special class of computational models that involve an agent learning from past experiences with the goal of identifying which actions in specific states lead to maximisation of rewarding outcomes[18]. Model-free RL models do not account for specialised task structures (such as a possible reversal in a PRL task) and instead learn based on the history of previous outcomes. Thus, fitting an RL model to data better approximates 'true' human behaviour compared to averaging scores to obtain a measure of behaviour (e.g., average proportion of perseverative errors) and enables improved insight into how learning evolves over time. In addition, compared to other classes of models, RL has been found to best capture PRL behaviour in marmosets[19] and has been widely used to model behaviour in studies investigating adolescent[20–22] and OCD samples[20,23].

In total, five RL models were fitted to data and compared to determine the best-fitting model. Model code was adapted from a prior study[23].

*Model 1*

Model 1 served to discern whether a simple RL model with two parameters best described the data and accounted for differences between groups. The model comprised a learning rate parameter ($\alpha$) and a reinforcement sensitivity parameter ($\tau$). A value function ($Q_t$) was assigned to each task stimulus representing the expected rewards associated with them. A high $Q_t$ denoted a higher chance of reward associated with a stimulus while a lower $Q_t$ indicated a reduced chance of a reward. $Q_t$ for each stimulus was updated on a trial-by-trial basis via prediction errors that represent differences between expected outcome, $Q_t$ (associated with previous trial's outcome), and actual current outcome, $R_t$. For example, if the expected outcome for a stimulus is 0, and selecting the stimulus on a given trial results in a reward, $Q_t$ for the specific stimulus would increase in value. Larger prediction errors thus led to greater updating of $Q_t$. On every trial (t), the value of free learning rate parameter $\alpha$ determined the extent to which $Q_t$ was adjusted according to the prediction error. Concretely, this was done according to the Rescorla-Wagner rule:

$Q_{k,t+1} = Q_{k,t} + \alpha(R_t - Q_{k,t})$ – Equation 1.1

Where k represents a specific stimulus (stimulus 1 or 2) and t represents the current trial. R would equal 1 following a rewarded outcome, and 0 following an unrewarded outcome. The term $R_t - Q_{k,t}$ represents the prediction error. $\alpha$ was able to vary between 0 and 1. Values of $\alpha$ closer to 1 indicate increased sensitivity to prediction errors, while lower values of $\alpha$ indicate low sensitivity to prediction errors.

$\tau$ (reinforcement sensitivity) is an inverse temperature parameter associated with the value functions assigned to each stimulus. This parameter was plugged into a softmax rule which was used to determine the probability (p) of choosing a stimulus k on trial t:

$p_{k,t} = \frac{\exp(\tau Q_{k,t})}{\sum_{i=1}^{k} \exp(\tau Q_{i,t})}$ – Equation 1.2

$\tau$ determined the extent to which participants' actions were driven by $Q_t$ associated with the chosen stimulus. A high $\tau$ leads to more exploitative behaviour whereby a participant chooses to mostly maximise their rewards (i.e., participants select the choice with the higher Q value more often). A low $\tau$ enables more exploratory behaviour (reduced selecting of the choice associated with the higher Q value).

*Model 2*

Model 2 was as Model 1 except $\alpha$ was fractionated to account for rewarding ($\alpha_{rew}$) and punishing outcomes ($\alpha_{pun}$) as there is evidence for different neural systems subserving learning from reward and punishment[24]. Hence, this model contained 3 free parameters, $\alpha_{rew}$, $\alpha_{pun}$, and $\tau$.

$Q_{k,t+1} = Q_{k,t} + \alpha_{rew}(R_t - Q_{k,t})$ – if R = 1 – Equation 1.3

$Q_{k,t+1} = Q_{k,t} + \alpha_{pun}(R_t - Q_{k,t})$ – if R = 0 – Equation 1.4

Higher values of $\alpha_{rew}$ leads to greater sensitivity to and quicker learning from positive prediction errors (i.e., rewarding trials) while higher values of $\alpha_{pun}$ results in expedited learning from negative prediction errors (punishing outcomes). The model was fit to data to investigate valence-specific learning. The softmax function including the $\tau$ parameter was implemented similarly to Model 1.

*Model 3*

Model 3 was identical to Model 2 but with the addition of $\tau_{stim}$ (stimulus stickiness), which is an inverse temperature parameter that reflects the tendency for a participant to respond to the same stimulus chosen in a previous trial regardless of feedback received. Greater values of $\tau_{stim}$ denote increased tendency to 'stick' to a choice while low values represent a tendency to switch away from the choice. Thus, $\tau_{stim}$ enabled us to account for perseverative behaviour. This parameter was added to the softmax function as follows:

$p_{k,t} = \frac{\exp(\tau Q_{k,t} + \tau_{stim} s_{k,t})}{\sum_{i=1}^{k} \exp(\tau Q_{i,t} + \tau_{stim} s_{i,t})}$ – Equation 1.5

$\tau_{stim}$ was multiplied by S which represents whether the stimulus chosen on the current trial was the same as the one chosen on the previous trial (1 if choices are repeated, 0 if not). If S = 1, $\tau_{stim}$ would influence the extent to which the choice is likely to be repeated. If S = 0, the function would reduce to the softmax function from Model 1. Thus, this model contained 4 parameters in total $\alpha_{rew}$, $\alpha_{pun}$, $\tau$, and $\tau_{stim}$.

*Model 4*

Model 4 was as Model 3 but with 3 parameters ($\alpha$, $\tau$, $\tau_{stim}$) as it contained a single learning rate controlling the adaptation of the value functions.

*Model 5*

Model 5 was distinct from the models described thus far. It was an experience-weighted attraction (EWA) model previously used by den Ouden et al. (2013)[25]. It contains 3 free parameters: $\varphi$ (phi), $\rho$ (rho), and $\beta$ (beta).

© 2021 Marzuki AA et al. *JAMA Network Open*.

The model served to decouple acquisition (pre-reversal) and reversal via the experience decay factor parameter ρ that enables the balance between previous experience and new information to increasingly tip in favour of past experiences. The 'experience weight' ($n_{c,t}$) of a current choice, *c*, reflects how often a stimulus has been chosen. It is updated according to ρ:

$n_{c,t} \leftarrow n_{c,t-1}\,\rho + 1$ – Equation 1.6

The intuition behind ρ is that over time experience accumulated during acquisition could make reversal more difficult leading to perseveration. ρ was allowed to range between 0 and 1. When ρ = 0, predictions are always driven by most recent outcomes, whereas when ρ = 1 all trials are weighted equally leading to perseveration of responses post-reversal.

The value function of a choice on every trial (similar to $Q_t$), $v_{c,t,}$ is updated according to the outcome (rewarded or unrewarded), $\lambda$, and the pay-off decay factor φ, which is equivalent to the learning rate in Model 1.

$v_{c,t} \leftarrow (v_{c,t-1}\,\varphi\, n_{c,t-1} + \lambda_{t-1}) \,/\, n_{c,t}$ – Equation 1.7

When $\rho = 0$, $n_{c,t}$ on every trial becomes 1 as per Equation 1.6, and therefore Equation 1.7 reduces to a standard Rescorla-Wagner model.

Equivalent to models described earlier, probability, *P*, of choices, *c*, was determined via a softmax function:

$P(c_{t+1} = i) = \frac{e^{\beta V(c=i,t+1)}}{\sum_j e^{\beta V(c=j,t+1)}}$ – Equation 1.8

Where the free inverse temperature parameter, $\beta$, controls the extent to which choices are made according to the value function V. Low values of $\beta$ lead to more exploratory choices while high values lead to choices that serve to maximise rewards.

*Model-Fitting and Parameter Estimation*

Models were fit to trial-by trial data using a hierarchical Bayesian approach, specifically by estimating the posterior distribution (distribution of estimated parameter values after model fitting) of the model parameters at the individual subject and group levels. This enabled estimating parameter distributions per group while controlling for inter-subject variability in behaviour.

At the top of the hierarchy, separate distributions were defined for OCD and CTL groups. Parameter estimations for each group were sampled from the following prior distributions:

$\alpha_{group}, \alpha_{group,rew}, \alpha_{group,pun}, \varphi_{group}, \rho_{group} \sim$ Beta (1.2,1.2)

$\tau_{group}, \beta_{group} \sim$ Gamma (4.82,0.88)

$\tau_{group,stim} \sim$ Normal(0,1)

Inter-subject variability, σ, was sampled from half-normal prior distributions, enabling estimates to be constrained to be positive.

$\sigma_{\alpha}, \sigma_{\alpha\text{-rew}}, \sigma_{\alpha\text{-pun}}, \sigma\tau_{\text{-stim}}, \sigma_{\varphi}, \sigma_{\rho} \sim$ half-Normal(0,0.05)

© 2021 Marzuki AA et al. *JAMA Network Open*.

$\sigma\tau$, $\sigma_{\beta}$~ half-Normal(0,1)

Subject-level parameters were sampled from normal distributions whose means were the group level parameter values and whose variances were from the inter-subject variability parameter values.

For example, in the case of $\alpha_{rew}$:

$\alpha_{rew,subject} = \alpha_{rew,group(subject)} + \sigma_{\alpha rew(subject)}$ – Equation 1.9

All priors were obtained from an earlier study which used identical models [23].

All models were fitted to data using Markov Chain Monte Carlo (MCMC) sampling implemented in RStan v. 2.21.1. Eight randomly-initialised MCMC chains were used. Convergence of chains was confirmed using the potential scale reduction statistic $\hat{R}$. A cut-off $\hat{R}$ value of 1.2[23] was used to check that the chains were well-mixed for each parameter.

In addition, model-fitting was repeated to explore the effects of medication on behaviour, specifically by specifying separate prior distributions for OCD, MED-, and MED+ (medicated patient).

*Parameter Recovery*

As these models had previously been fitted to data obtained from a PRL task with several reversals, we conducted parameter recovery to verify the validity of the winning model (see Model Comparisons section below for how we compared models) and that parameter values were meaningful (and not occurring by chance)[26]. The winning model was first used to simulate synthetic data from 100 'participants'. The free parameters were replaced with the mean fitted parameter values per group estimated from fitting the model to actual human data. We then ascertained whether the true parameter values could be recovered by fitting the winning model to the simulated data and checking whether the true and generated parameter values fell within their corresponding 95% highest density interval (HDI) – see Group Differences section below for more details about HDI.

**Computational Models for WCST**

The main reinforcement learning model fitted to WCST data was originally conceptualised as a sequential learning model by Bishara et al. (2010)[27]. This modelling approach has been used to successfully reveal implicit cognitive strategies employed by various neuropsychiatric patient groups, among them individuals with substance dependence[27] (Bishara et al., 2010), prefrontal cortex lesions[12] and schizophrenia[28]. However, the model has yet to be implemented in research involving patients with OCD. The model contained 4 free parameters, namely reward rate (*r*, how quickly attention weights change to rewarding feedback), punishment rate (*p*, how quickly attention weights change to punishing feedback), decision consistency (*d*, how much deck choice is influenced by attention weights), attentional focusing (*f*, only important on trials with ambiguous feedback and represents the degree to which the update is focused only on the category/rule with the largest attention weight).

The dependent variables fed into the model are 1) an outcome variable which represents whether a trial was rewarded or not (1 or 0) and 2) a matching matrix which quantifies which categories (colour, number, or shape) associated with

© 2021 Marzuki AA et al. *JAMA Network Open*.

a chosen deck match the test card. For instance, if the chosen deck matched the test card based on colour but not number or shape, the matching matrix, m, for that trial would be defined as

$$m = \begin{bmatrix} 1 \\ 0 \\ 0 \end{bmatrix}$$ – Equation 2.1

The model calculates the probabilities associated with choosing each deck as a function of attention weights (a), which represents the weight/value given to each category per trial. The matrix elements of the attention signal always sum to one. It was assumed that for each participant's first trial, the attention weights are divided evenly between categories:

$$a = \begin{bmatrix} .333.. \\ .333.. \\ .333.. \end{bmatrix}$$ – Equation 2.2

Attention weights are updated using a feedback signal (s), which represents whether the categories were rewarded or not. For example, in the case where a chosen deck matches the test card based on only colour and nothing else, and the trial is rewarded, the feedback signal would look like this,

$$s = \begin{bmatrix} 1 \\ 0 \\ 0 \end{bmatrix}$$ – Equation 2.3

with each element of s representing colour, number, and shape respectively. The current attention weights are updated based on the feedback signal according to the following equations:

$\mathbf{a}_{t+1}\,|\text{rewarded}_t = (1\text{-}r)\mathrm{a}_t + r\mathbf{s}$ - if trial was rewarded – Equation 2.4

$\mathbf{a}_{t+1}\,|\text{punished}_t = (1\text{-}p)\mathrm{a}_t + p\mathbf{s}$ - if trial was punished – Equation 2.5

where t refers to the current trial. Parameters *r* and *p* determine how rapidly attention weights alter based on rewarding and punishing feedback signals respectively.

In the example above, the feedback is unambiguous as the chosen deck matches the test card on only one category. However, in some cases where more than one category is matched (for example, both colour and shape), the feedback signal relies on the free parameter *f* to modulate how focused or wide the attention is for each category's feedback. When *f* approaches 0, attention is split evenly between the matching categories:

$$s = \begin{bmatrix} 0.5 \\ 0.5 \\ 0 \end{bmatrix}$$ – Equation 2.6

As *f* increases, the feedback signal is split proportionally between current attention weights. For example, if the attention weight for colour is higher than shape, the feedback signal would follow suit and perhaps be represented by:

$$s = \begin{bmatrix} 0.75 \\ 0.25 \\ 0 \end{bmatrix}$$ – Equation 2.7

© 2021 Marzuki AA et al. *JAMA Network Open*.

The following equations represent how the feedback signal is modulated by the attention weights and the matching matrix.

$s_t|\text{reward} = \frac{\mathbf{m_t a_t^f}}{\Sigma \mathbf{m_t a_t^f}}$ – Equation 2.8

$s_t|\text{punish} = \frac{(1-\mathbf{m_t})\mathbf{a_t^f}}{\Sigma(1-\mathbf{m_t})\mathbf{a_t^f}}$ – Equation 2.9

When outcome on the current trial is correct, the feedback signal is computed only with the matching attention weights, and when the outcome is incorrect, only the non-matching attention weights contribute to the feedback signal.

Finally, the probability of choosing a specific deck is defined as

$P = \frac{\mathbf{m}_t' \boldsymbol{a}_t^d}{\Sigma \boldsymbol{a}_t^d}$ – Equation 2.10

where the *d* parameter influences the predicted probability of choosing a deck per trial. As *d* becomes lower, choices become more random and less dependent on attention weights (more exploratory). As *d* becomes higher, choices are heavily constrained by attention weights (more exploitative). $\mathbf{m}_t'$ is simply the matching matrix, $\mathbf{m}_t$ ,transposed to enable matrix multiplication (dot product) with $\boldsymbol{a}_t$.

The full model described above with 4 free parameters was compared to 3 degenerate models. Each degenerate model had one parameter fixed to assess the contribution of each parameter to capturing behaviour on the task.

The 1st alternative model (RPD0) fixed the *f* parameter to be 0, the 2nd alternative model (RP1F) fixed *d* to be 1, and the final model (RRDF) assumed a single common learning rate for both reward and punishment.

Model code was adapted from a prior study[27].

*Model-Fitting and Parameter Estimation*

As with the PRL models, WCST models were fit to trial-by trial behavioural data using hierarchical Bayesian estimation by estimating the posterior distribution of the parameters at the individual subject- and group- levels. At the group level, Uniform (0,1) distributions were used as priors for the *r* and *p* parameters, while Uniform (0,5) distributions were used for *d* and *f* parameters. Inter-subject variability for *r* and *p* were sampled from Half-normal (0,0.05) distributions, while inter-subject variability for *d* and *f* were sampled from Half-normal (0,1) distributions. Individual subject parameters were sampled from normal distributions, and the mean and variance of the prior distributions were sampled from the group-level and inter-subject variability distributions.

Computation of the posteriors were conducted using Markov MCMC sampling using JAGS software[29]. Four randomly initialised MCMC chains were run during model-fitting. Convergence was checked using the statistic $\hat{R}$.

Lastly, model-fitting was repeated to explore the effects of medication on behaviour. Group differences between CTL, MED-, and MED+ were analysed. Parameter recovery was not run here as the winning model had already been fully validated in a previous study[12].

### Model Comparison

Models for both tasks were compared using bridge sampling via the "bridgesampling" R package[30]. This method enables selection of the best-fitting model by accounting for the prior probability and marginal likelihood of each model (the likelihood of the data given a specific model). The marginal likelihood is calculated via the product of 1) the likelihood of the data given a fitted model (how well does the model fit the data) and 2) the probability of parameters given the model, which penalises over-complex models and guards against overfitting. We assumed equal prior probability for each model per task.

### Group Differences

Posterior distributions of parameters from both tasks' winning models were interpreted using the 95% and 90% highest posterior density interval (HDI), also known as the Bayesian credible interval. All values within the interval have a higher probability density (i.e. higher credibility) than any value outside the HDI. Parameter comparisons between groups were calculated by subtracting the posterior distributions of the CTL group-parameters from the posterior distributions of the OCD group-parameters, generating the group mean differences per parameter. This procedure was also done for MED- vs. MED+ vs. CTL. The 95% and 90% HDIs of the posterior distribution for the group mean differences were calculated and inspected to check whether they reliably included zero (indicating no difference between groups)

Multiple comparisons corrections were not applied since a strength of Bayesian hierarchical models is that they make comparisons more conservative through "shrinkage" of estimates drawn from a higher-level distribution. This makes estimates lie closer together, leading to a higher likelihood for intervals (HDIs) to include 0[31].

© 2021 Marzuki AA et al. *JAMA Network Open*.

**Standard PRL Results**

Visualisations for the PRL group results are shown in eFigures 1 and 2. The mixed regression analyses were repeated controlling for age, gender, and IQ. These results are summarised in eTable 1.

## eTable 1. Standard Mixed Regression Results for PRL Covarying for Age, Gender, and IQ

| **Dependent Variable** | **Independent Variable** | **Estimate** | **p** | **BH-adjusted p** | **95% CI** | **Test used** |
|---|---|---|---|---|---|---|
| p(Correct) | Age<br>Gender<br>IQ[a]<br>Group<br>Phase[a]<br>Group x Phase[a] | 0.16<br>-0.31<br>0.03<br>-0.18<br>-1.65<br>-0.94 | .12<br>.14<br>.003<br>.45<br><.0001<br><.0001 | .35<br>.39<br>.01<br>.45<br><.0001<br><.0001 | -0.041 – 0.36<br>-0.71 – 0.10<br>0.01 – 0.04<br>-0.67 – 0.30<br>-1.90 – -1.40<br>-1.30 – -0.61 | Binomial Mixed Model |
| RT | Age<br>Gender<br>IQ<br>Group<br>Phase[a]<br>Group x Phase | 16.00<br>32.54<br>-5.32<br>81.14<br>-60.52<br>-73.02 | .66<br>.66<br>.10<br>.29<br>.04<br>.09 | .72<br>.66<br>.12<br>.38<br>.04<br>.09 | -54.53 – 86.52<br>-109.53 – 174.61<br>-11.47 – 0.83<br>-64.74 – 227.03<br>-118.24 – -2.80<br>-156.29 – 10.26 | Mixed Linear Model |
| p(Switching to SNF) | Age<br>Gender<br>IQ[a]<br>Group<br>Phase[a]<br>Group x Phase[a] | -0.17<br>-0.64<br>-0.07<br>0.88<br>0.50<br>0.82 | .58<br>.30<br>.13<br>.16<br>.0001<br><.0001 | .72<br>.39<br>.02<br>.38<br>.0001<br><.0001 | -0.76 – 0.43<br>-1.86 – 0.57<br>-0.12 – -0.01<br>-0.34 – 2.09<br>0.25 – 0.75<br>0.50 – 1.15 | Binomial Mixed Model |
| p(Staying to VPF) | Age<br>Gender<br>IQ[a]<br>Group<br>Phase[a]<br>Group x Phase[a] | 0.37<br>-0.53<br>0.06<br>-0.54<br>-0.95<br>-1.68 | .14<br>.29<br>.42<br>.31<br><.0001<br><.0001 | .35<br>.39<br>.01<br>.38<br><.0001<br><.0001 | -0.12 – 0.85<br>-1.51 – 0.45<br>0.02 – 0.10<br>-1.57 – 0.49<br>-1.28 – -0.62<br>-2.11 – -1.25 | Binomial Mixed Model |
| **Dependent Variable** | **Independent Variable** | **Estimate** | **P** | **BH-adjusted p** | **95% CI** | **Test Used** |
| p(Perseverative Error) | Group<br>Age<br>IQ<br>Gender | 0.73<br>-0.06<br>-0.02<br>0.31 | .02<br>.72<br>.23<br>.31 | .10<br>.72<br>.39<br>.23 | 0.56 – 0.90<br>-0.14 – 0.03<br>-0.02 – - 0.01<br>0.14 – 0.48 | Binomial Model |

Key- [a]significant at p<.05; df: degrees of freedom; BH: Benjamini-Hochberg correction; CI: confidence interval; p(Correct): proportion correct choices; RT: mean reaction time; p(Switching following SNF): proportion of switching in response to spurious (false) negative feedback; p(Staying following VPF): proportion of staying in response to veridical (true) positive feedback; p(Perseverative Error): proportion of perseverative errors (only during reversal phase). Original p-values and BH corrected p-values are reported.

© 2021 Marzuki AA et al. *JAMA Network Open*.

## eFigure 1. Group Comparisons (CTL vs OCD) Across All PRL Measures

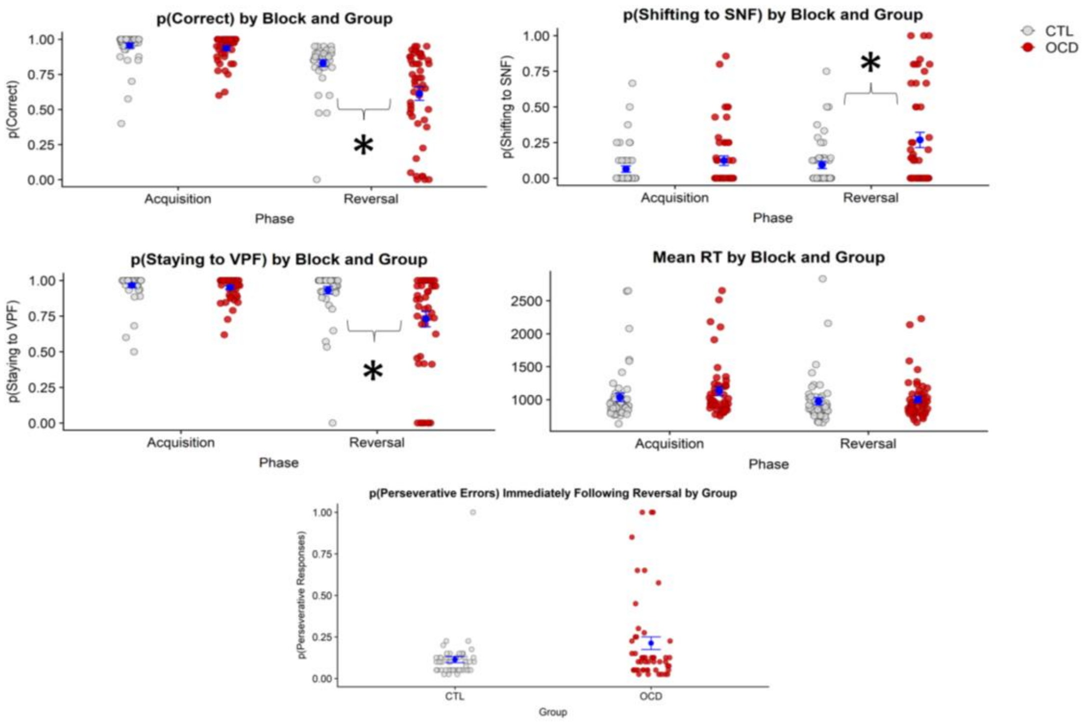


*p<.05, adjusted using Benjamini-Hochberg Correction. Error bars represent standard errors. Each point represents one subject.

Summary statistics and regression results comparing CTL with MED- and MED+ on PRL task measures are summarised in eTables 2, 3, and 4 (also see eFigure 2 for visualisation). The 3 groups were matched for age, IQ, and gender. However, post-hoc Dunn tests with Bonferroni correction revealed that MED+ and MED- had enhanced depression (MED- > CTL, $p$ = .001; MED+ > CTL, $p$ < .0001; MED+ = MED-, $p$ = .63), anxiety (MED- > CTL, $p$ < .0001; MED+ > CTL, $p$ < .0001; MED+ = MED-, $p$ = .68), and obsessive-compulsivity scores (MED- > CTL, $p$ <.0001; MED+ > CTL, $p$<.0001; MED+ = MED-, $p$ = 1.00) compared to CTL. There were no differences in these ratings between MED+ and MED- (all $p$ > .05) (see eTable 2).

When analysing task measures, we found significant Group-by-Phase interactions for p(Correct) [Coefficient estimate= -0.53,95% CI: - 0.73 – -0.33, BH-adjusted p<.0001], p(Shifting following SNF) [Coefficient estimate= 0.42, 95% CI:0.23 – 0.60, BH-adjusted p<.0001], and p(Staying following VPF) [Coefficient estimate=-0.93, 95% CI:-1.18 - -0.68, BH-adjusted p<.0001]. Other task measures showed no significant Group or Group-by-Phase interaction effects ($p$ > .05). See eTables 3 and 4 for task summary statistics and analyses for CTL vs MED- vs MED+.

As a result of the significant interactions, we conducted post-hoc comparisons between groups within each Phase. These analyses revealed that during the Reversal phase, compared to CTL, MED- and MED+ made significantly less correct responses (MED- < CTL: z-ratio = 4.67, 95% CI: 0.50 – 2.2; MED+ < CTL: z-ratio = 4.48, 95% CI: 0.39 – 1.88; MED- = MED+: z-ratio = -0.67, 95% CI: -1.14 – 0.72) and stayed less following VPF (MED- < CTL: z-ratio = 4.08, 95% CI: 0.82 – 5.08 ; MED+ < CTL: z-ratio = 4.17, 95% CI: 0.79 – 4.57; MED- = MED+: z-ratio =-0.36, 95% CI: -2.48 – 1.93). However, group differences between medication groups and CTL for p(switching following SNF)

© 2021 Marzuki AA et al. *JAMA Network Open*.

did not survive Bonferroni correction (MED- = CTL: z-ratio = -2.46, 95% CI: -4.39 – 0.38; MED+ = CTL: z-ratio = -2.73, 95% CI: -4.05 – 0.15; MED- = MED+: z-ratio = 0.06, 95% CI: -2.50 – 2.61).

© 2021 Marzuki AA et al. *JAMA Network Open*.

## eTable 2. Summary Statistics for Demographic and Clinical Measures (CTL vs MED- vs MED+)

| | CTL (n = 53) | MED- (n=20) | MED+ (n=30) | STATISTIC | PAIRWISE COMPARISONS |
|---|---|---|---|---|---|
| GENDER (F:M) | 30:23 | 15:5 | 14:16 | χ2 (2)=3.96; p=.14 | - |
| AGE | Median =16.41 IQR = 14.8 - 18.0 | Median=16.63 IQR = 15.0 – 17.5 | Median=16.68 IQR = 15.4 – 18.1 | χ2 (2)=0.44; p= .80 | - |
| WASI-II (IQ)[b] | 109.11 (10.79) | 105.0 ± 11.85 | 107.57 ± 12.34 | F(2,99) = .92; p= .40 | - |
| BDI[a] | 46.81 (6.43) | Median=57.5 IQR = 49.8 – 61.5 | Median=62.5 IQR = 53.5 – 67.8 | χ2 (2)=36.20; p= 1.38e-08 | MED- & MED+ >CTL MED- = MED+ |
| BAI[a] | 48.04 (7.09) | Median = 61 IQR = 49.5 – 67.5 | Median = 65 IQR = 58.3 – 68.8 | χ2 (2)=42.74; p=5.24e-10 | MED- & MED+ >CTL MED- = MED+ |
| OCI[a] | Median = 8 IQR = 4 - 14 | Median = 17.5 IQR = 19.3 – 36.8 | Median=17.75 IQR = 18.0 – 35.8 | χ2 (2)=57.31; p < .0001 | MED- & MED+ >CTL MED- = MED+ |
| CY-BOCS[b] | N/A | Median = 24 IQR = 20.5 – 26.5 | Median = 23 IQR = 20 - 26 | Z = 0.48, p=.63 | - |

Key: CTL: Control Group; MED-: Unmedicated OCD Group; MED+: Medicated OCD Group WISC-IV: Wecshler's Abbreviated Scale of Intelligence – II; IQ: Intelligence Quotient; BDI: Beck's Depression Inventory (t-scored); BAI: Beck's Anxiety Inventory (t-scored); OCI: Obsessive-Compulsive Inventory; CY-BOCS: Children's Yale-Brown Obsessive-Compulsive Scale. [a]significant at p<.05; [b]missing data from one MED- participant. Means and Standard Deviations M(SD) reported for normally distributed data. Median and interquartile range (IQR) reported for non-normally distributed data.

© 2021 Marzuki AA et al. *JAMA Network Open*.

## eTable 3. Summary Statistics for PRL Task Measures (CTL vs MED- vs MED+)

| | ACQUISITION | | | REVERSAL | | |
|---|---|---|---|---|---|---|
| **Dependent Variable** | **CTL** | **MED-** | **MED+** | **CTL** | **MED-** | **MED+** |
| p(perseverative error) | - | - | - | 0.11 (0.13) | 0.24 (0.29) | 0.19 (0.25) |
| RT | 1035.92 (401.98) | 1148.08 (511.15) | 1117.02 (373.29) | 975.41 (355.06) | 1005.78 (353.64) | 997.55 (281.18) |
| p(Correct) | 0.96 (0.11) | 0.93 (0.10) | 0.94 (0.097) | 0.83 (0.16) | 0.60 (0.29) | 0.63 (0.33) |
| p(Switching following SNF) | 0.064 (0.14) | 0.11 (0.21) | 0.13 (0.22) | 0.094 (0.16) | 0.27 (0.36) | 0.27 (0.34) |
| p(Staying following VPF) | 0.97 (0.098) | 0.95 (0.061) | 0.95 (0.093) | 0.93 (0.166) | 0.74 (0.31) | 0.73 (0.37) |

Key- MED-: unmedicated OCD group; MED+: medicated OCD group; p(Correct): proportion correct choices; mean RT: reaction time; p(Switching following SNF): proportion of switching in response to spurious (false) negative feedback; p(Staying following VPF): proportion of staying in response to veridical (true) positive feedback; p(Perseverative Error): proportion of perseverative errors (only during reversal phase).

© 2021 Marzuki AA et al. *JAMA Network Open*.

**eTable 4. Standard Regression Results for PRL Without Covariates (CTL vs MED- vs MED+)**

| **Dependent Variable** | **Independent Variable** | **Estimate** | **p** | **BH-adjusted p** | **95% CI** | **Test used** |
|---|---|---|---|---|---|---|
| p(Correct) | Group<br>Phase[a]<br>Group x Phase[a] | -0.08<br>-1.22<br>-0.53 | .60<br><.0001<br><.0001 | .60<br><.0001<br><.0001 | -0.38 – 0.22<br>-1.63 – -0.83<br>-0.73 – -0.33 | Binomial Mixed Model |
| RT | Group<br>Phase<br>Group x Phase | 44.66<br>-35.87<br>-32.48 | .30<br>.46<br>.19 | .37<br>.50<br>.19 | -39.10 – 128.41<br>-130.61 – 58.88<br>-80.38 – 15.42 | Mixed Linear Model |
| p(Switching following SNF) | Group<br>Phase<br>Group x Phase[a] | 0.59<br>0.24<br>0.42 | .10<br>.24<br><.0001 | .25<br>.48<br><.0001 | -0.11 – 1.30<br>-0.16 – 0.63<br>0.23 – 0.60 | Binomial Mixed Model |
| p(Staying following VPF) | Group<br>Phase<br>Group x Phase[a] | -0.47<br>-0.18<br>-0.93 | .17<br>.50<br><.0001 | .28<br>.50<br><.0001 | -1.14 – -0.20<br>-0.71 – 0.35<br>-1.18 – -0.68 | Binomial Mixed Model |
| Dependent Variable | Independent Variable | Estimate | p | BH-Adjusted p | 95% CI | Test Used |
| p(Perseverative Errors) | Group | 0.32 | .06 | .25 | -0.017 – 0.65 | Binomial Model |

Key- [a]significant at p<.05; MED-: unmedicated OCD group; MED+: medicated OCD group; M: mean; SD: standard deviation; df: degrees of freedom; BH: Benjamini-Hochberg Correction; CI: confidence interval; p(Correct): proportion correct choices; RT: mean reaction time; p(Switching following SNF): proportion of switching in response to spurious (false) negative feedback; p(Staying following VPF): proportion of staying in response to veridical (true) positive feedback; p(Perseverative Error): proportion of perseverative errors (only during reversal phase). Original p-values and BH corrected p-values are reported.

**eTable 5. Standard Regression Results for PRL Covarying for Age, Gender, and IQ (CTL vs MED- vs MED+)**

| Dependent Variable | Independent Variable | Estimate | p | BH-adjusted p | 95% CI | Test used |
|---|---|---|---|---|---|---|
| p(Correct) | Age<br>Gender<br>IQ[a]<br>Group<br>Phase[a]<br>Group x Phase[a] | 0.17<br>-0.25<br>0.03<br>-0.05<br>-1.20<br>-0.52 | .11<br>.24<br>.002<br>.73<br><.0001<br><.0001 | .32<br>.55<br>.007<br>.73<br><.0001<br><.0001 | -0.04 – 0.38<br>-0.67 – 0.17<br>0.01 – 0.05<br>-0.34 – 0.23<br>-1.61 – -0.80<br>-0.72 – -0.32 | Binomial Mixed Model |
| RT | Age<br>Gender<br>IQ<br>Group<br>Phase<br>Group x Phase | 15.77<br>29.92<br>-5.42<br>37.02<br>-36.86<br>-33.10 | .67<br>.69<br>.09<br>.40<br>.45<br>.18 | .72<br>.69<br>.11<br>.53<br>.45<br>.18 | -56.14 – 87.68<br>-115.05 – 174.88<br>-11.64 – 0.81<br>-48.01 – 122.04<br>-131.63 – 57.91<br>-81.02 – 14.82 | Mixed Linear Model |
| p(Switching following SNF) | Age<br>Gender<br>IQ<br>Group<br>Phase<br>Group x Phase[a] | -0.16<br>-0.51<br>-0.05<br>0.41<br>0.17<br>0.41 | .58<br>.44<br>.03<br>.22<br>.40<br><.0001 | .72<br>.55<br>.06<br>.53<br>.45<br><.0001 | -0.73 – -0.41<br>-1.66 – 0.64<br>-0.10 – -0.004<br>-0.24 – 1.07<br>-0.23 – 0.56<br>0.23 – 0.59 | Binomial Mixed Model |
| p(Staying following VPF) | Age<br>Gender<br>IQ<br>Group<br>Phase<br>Group x Phase[a] | 0.39<br>-0.40<br>0.07<br>-0.25<br>-0.23<br>-0.89 | .13<br>.42<br>.003<br>.42<br>.41<br><.0001 | .32<br>.55<br>.007<br>.53<br>.45<br><.0001 | -0.11 – 0.88<br>-1.40 - 0.61<br>0.02 – 0.11<br>-0.84 – 0.35<br>-0.75 – 0.30<br>-1.14 – -0.64 | Binomial Mixed Model |
| **Dependent Variable** | **Independent Variable** | **Estimate** | | **p** | **2.5%** | **Test Used** |
| p(Perseverative Errors) | Group<br>Age<br>IQ<br>Gender | 0.30<br>-0.057<br>-0.017<br>0.26 | .09<br>.71<br>.20<br>.42 | .43<br>.72<br>.20<br>.55 | -0.04 – 0.64<br>-0.37 – 0.25<br>-0.04 – 0.009<br>-0.36 – 0.87 | Binomial Mixed Model |

Key- [a]significant at p<.05; MED-: unmedicated OCD group; MED+: medicated OCD group; M: mean; SD: standard deviation; df: degrees of freedom; BH: Benjamini-Hochberg Correction; CI: confidence interval; p(Correct): proportion correct choices; RT: mean reaction time; p(Switching following SNF): proportion of switching in response to spurious (false) negative feedback; p(Staying following VPF): proportion of staying in response to veridical (true) positive feedback; p(Perseverative Error): proportion of perseverative errors (only during reversal phase). Original p-values and BH corrected p-values are reported.

© 2021 Marzuki AA et al. *JAMA Network Open*.

## eFigure 2. Group Comparisons (CTL vs MED- vs MED+) Across All PRL Measures

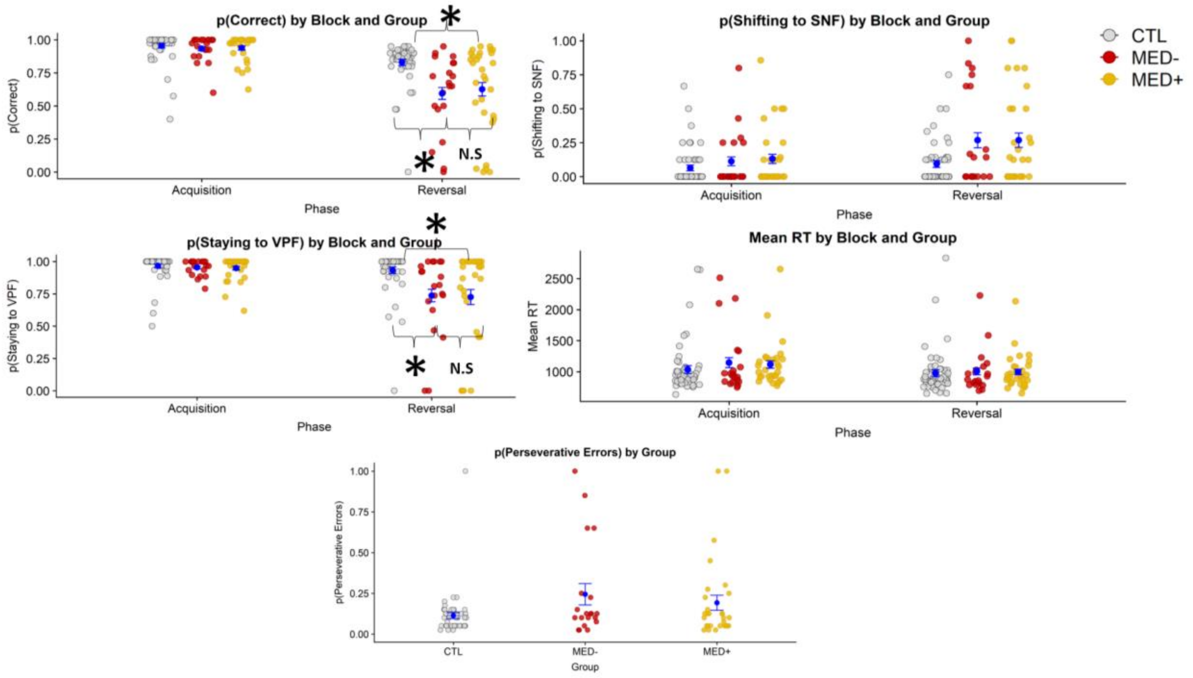


Key: N.S.: No significant differences; *p<.05, adjusted using Benjamini-Hochberg Correction. Error bars represent standard errors. Each point represents one subject.

### Standard WCST Results

The regression analyses comparing CTL and OCD revealed a significant effect of group on RT and unique error measures (see eTable 6 and eFigure 3). OCD showed higher RTs [CTL: 1420.49 (279.72); OCD:1580.13 (301.478); coefficient estimate = 159.64, 95% CI: 20.47 – 298.80] and increased unique errors compared to CTL [CTL: 0.0014 (0.0045); OCD: 0.0047 (0.0047); coefficient estimate = 1.23, 95% CI: 0.16 – 2.44]. However, these comparisons ceased to be significant following Benjamini-Hochberg corrections [RT: BH-adjusted $p = .12$; p(unique errors): BH-adjusted $p = .12$]. All other measures also showed no significant group differences ($p > .05$). There were equally no significant differences on any WCST measures when controlling for age, gender, and IQ (all $p > .05$). See eTables 6 and 7, and eFigure 3.

© 2021 Marzuki AA et al. *JAMA Network Open*.

## eTable 6. Standard Regression Results for WCST Without Covariates (CTL vs OCD)

| Dependent Variable | Independent Variable | Estimate | p | BH-adjusted p | 95% CI | Test Used |
|---|---|---|---|---|---|---|
| Number of sets completed | Group | -0.44 | .29 | .34 | -1.26 – 0.38 | Linear Model |
| p(Perseverative errors) | Group | 0.54 | .54 | .54 | -0.14 – 0.26 | Binomial Model |
| p(Non perseverative errors) | Group | 0.30 | .12 | .21 | -0.07 – 0.66 | Binomial Model |
| Mean RT | Group | 159.64 | .03 | .12 | 20.47 – 298.80 | Linear Model |
| Failure to maintain set | Group | 0.55 | .07 | .17 | -0.05 – 1.15 | Linear Model |
| Number of trials needed to complete first set | Group | 3.34 | .26 | .34 | -2.50 – 9.19 | Linear Model |
| p(Unique Errors) | Group | 1.23 | .03 | .12 | 0.16 – 2.44 | Binomial Model |

Key- CTL: Control group; OCD: Patient group; M: mean; SD: standard deviation; df: degrees of freedom; BH: Benjamini-Hochberg Correction; CI: confidence interval; p(): proportion. Original p-values and BH corrected p-values are reported.

© 2021 Marzuki AA et al. *JAMA Network Open*.

**eTable 7. Standard Regression Results for WCST Covarying for Age, Gender, and IQ (CTL vs OCD)**

| Dependent Variable | Independent Variable | Estimate | p | BH-adjusted p | 95% CI | Test Used |
|---|---|---|---|---|---|---|
| Number of sets completed | Group<br>Gender<br>Age[a]<br>IQ | -0.32<br>-0.64<br>0.61<br>0.03 | .41<br>.11<br>.002<br>.03 | .48<br>.59<br>.006<br>.07 | -1.09 – 0.45<br>-1.43 – 0.14<br>0.24 – 1.00<br>0.003 – 0.07 | Linear Regression |
| p(Perseverative errors) | Group<br>Gender<br>Age[a]<br>IQ[a] | 0.04<br>0.08<br>-0.13<br>-0.01 | .68<br>.37<br>.004<br>.0004 | .68<br>.59<br>.008<br>.003 | -0.14 – 0.21<br>-0.09 – 0.25<br>-0.21 – -0.04<br>-0.02 – -0.006 | Binomial Model |
| p(Non perseverative errors) | Group<br>Gender<br>Age[a]<br>IQ | 0.24<br>0.12<br>-0.20<br>-0.007 | .17<br>.52<br>.02<br>.36 | .31<br>.61<br>.03<br>.51 | -0.11 – 0.59<br>-0.24 – 0.47<br>-0.37 – -0.03<br>-0.02 – 0.007 | Binomial Model |
| Mean RT | Group<br>Gender<br>Age[a]<br>IQ[a] | 128.44<br>51.25<br>-139.68<br>-6.67 | .04<br>.42<br><.0001<br>.01 | .14<br>.59<br>.0001<br>.03 | 5.55 – 251.33<br>-74.43 – 176.93<br>-199.85 – -79.14<br>-11.69 – -1.65 | Linear Regression |
| Failure to maintain set | Group<br>Gender<br>Age[a]<br>IQ | 0.54<br>0.36<br>-0.30<br>-0.02 | .07<br>.24<br>.04<br>.11 | .17<br>.59<br>.04<br>.20 | -0.06 – 1.13<br>-0.24 – 0.97<br>-0.59 – -0.02<br>-0.04 – 0.004 | Linear Regression |
| Number of trials needed to complete first set | Group<br>Gender<br>Age[a]<br>IQ | 2.65<br>-1.00<br>-3.34<br>-0.049 | .27<br>.74<br>.02<br>.68 | .48<br>.74<br>.03<br>.80 | -3.26 – 8.56<br>-7.05 – 5.04<br>-6.23 – -0.44<br>-0.29 – 0.19 | Linear Regression |
| p(Unique Errors) | Group<br>Gender<br>Age[a]<br>IQ | 1.19<br>-0.43<br>-0.66<br>0.002 | .02<br>.41<br>.005<br>.91 | .14<br>.59<br>.008<br>.91 | 0.23 – 2.26<br>-1.51 – 0.54<br>-1.11 – -0.22<br>-0.037 – 0.04 | Binomial Model |

Key- [a]significant at p<.05; CTL: Control group; OCD: Patient group; M: mean; SD: standard deviation; df: degrees of freedom; BH: Benjamini-Hochberg Correction; CI: confidence interval; p(): proportion. Original p-values and BH corrected p-values are reported.

© 2021 Marzuki AA et al. *JAMA Network Open*.

## eFigure 3. Group Comparisons (CTL vs OCD) Across All WCST Measures

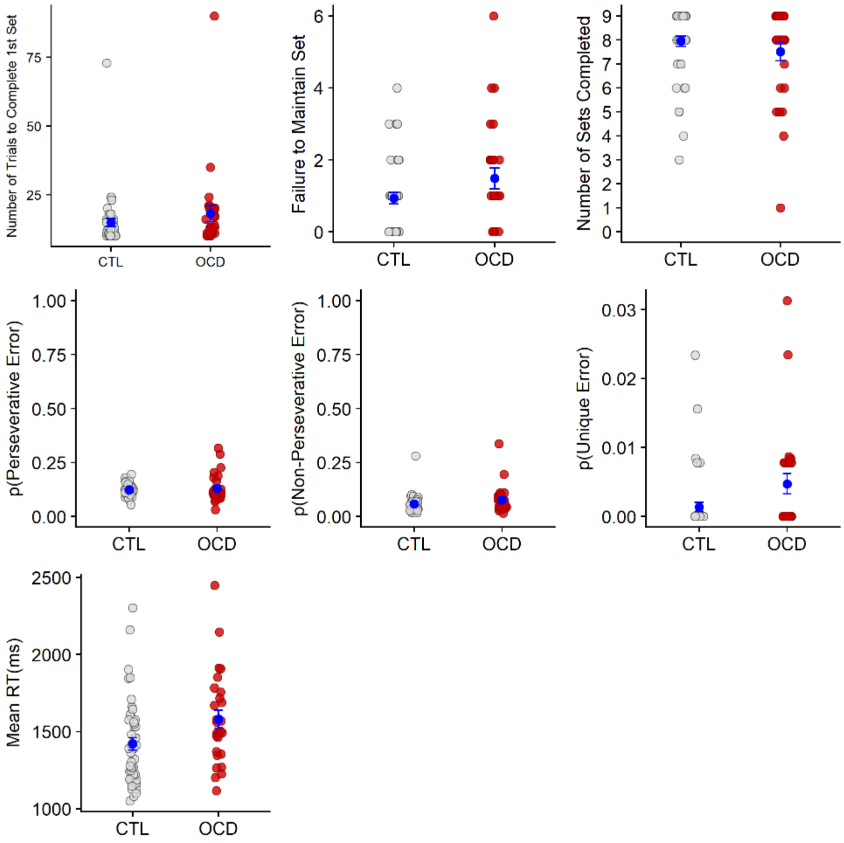


No significant differences between CTL and OCD on any measures when controlling for multiple corrections using the Benjamini-Hochberg procedure. Error bars represent standard errors. Each point represents one subject.

When dividing OCD into MED- and MED+, we verified that the groups were well matched for age, IQ, and gender. MED- and MED+ showed significantly elevated OCI, BDI, and BAI scores compared to CTL (see eTable 8). Summary scores per WCST outcome measure per group are reported in eTable 9, results of regression analyses are reported in eTable 10, and regression results with age, IQ, gender as covariates are in eTable 11. Other group differences aside from RT and p(unique errors) were insignificant ($p > .05$).

See eFigure 4 for visualisations.

## eTable 8. Summary Statistics for Demographic and Clinical Measures (CTL vs MED- vs MED+)

| | CTL (n = 46) | MED- (n = 16) | MED+ (n = 11) | STATISTIC | PAIRWISE COMPARISONS |
|---|---|---|---|---|---|
| SEX (F:M) | 28/18 | 13/3 | 5/6 | χ2(2) =3.83 ; *p* = 0.15 | - |
| AGE | Median = 17.17 IQR = 16.3 - 17.6 | Median=16.28 IQR = 15.6 – 17.2 | Median=15.83 IQR = 15.0 – 16.8 | χ2(2) = 3.67 ; *p* = 0.16 | - |
| WASI-II (IQ)[b] | 107.61 (11.62) | 107.27 (13.64) | 106.9 (14.96) | F(2,69) = 0.02; *p* = 0.98 | - |
| BDI[a] | 46.46 (5.27) | Median= 60 IQR = 54.5 – 67.0 | Median=58 IQR = 53.5 – 64.0 | χ2(2) = 30.03; *p* < .001 | CTL < MED- & MED+ MED- = MED+ |
| BAI[a] | Median = 46 IQR = 44 - 50 | Median=62.5 IQR = 59.0 – 70.8 | Median= 64 IQR = 58.5 – 68.5 | χ2(2) =47.44 ; *p* < .001 | CTL < MED- & MED+ MED- = MED+ |
| OCI[a] | Median = 6.5 IQR = 4 - 11 | Median=30.5 IQR = 23.5 – 36.3 | Median=28 IQR = 25.0 – 35.5 | χ2(2) =40.28; *p* < .001 | CTL < MED- & MED+ MED- = MED+ |
| CY-BOCS[b] | N/A | 24.47 (4.92) | 22.45 (4.70) | *t*(24) = 1.05; *p* = 0.30 | N/A |

Key: CTL: Control Group; MED-: Unmedicated OCD group; MED+: Medicated OCD group; WASI-II: Wecshler's Abbreviated Scale of Intelligence – II; IQ: Intelligence Quotient; BDI: Beck's Depression Inventory (t-scored); BAI: Beck's Anxiety Inventory (t-scored); OCI: Obsessive-Compulsive Inventory; CY-BOCS: Child Yale-Brown Obsessive-Compulsive Scale; mg: milligrams. [a]significant at p<.05; [b]missing data from one MED- participant. Means and Standard Deviations M(SD) reported for normally distributed data. Median and interquartile range (IQR) reported for non-normally distributed data.

© 2021 Marzuki AA et al. *JAMA Network Open*.

# eTable 9. Scores per Outcome Measure per Group (CTL vs MED- vs MED+)

| **Outcome Measures** | **CTL (n=46)** | **MED- (n=16)** | **MED+ (n=11)** |
|---|---|---|---|
| Number of sets completed | 7.96 (1.48) | 7.44 (2.22) | 7.63 (1.80) |
| p(Perseverative errors) | 0.12 (0.03) | 0.13 (0.08) | 0.13 (0.05) |
| p(Non-perseverative errors) | 0.06 (0.04) | 0.08 (0.08) | 0.06 (0.03) |
| p(Unique errors) | 0.001 (0.004) | 0.002 (0.004) | 0.008 (0.01) |
| Mean RT | 1420.49 (279.71) | 1471.42 (212.81) | 1738.25 (349.23) |
| Failure to maintain set | 0.94 (1.06) | 1.56 (1.55) | 1.36 (1.50) |
| Number of trials needed to complete first set | 14.80 (9.58) | 20.31 (19.66) | 15.00 (5.08) |

Mean(Standard Deviation) reported. Key: CTL: Control Group; MED-: Unmedicated OCD group; MED+: Medicated OCD group; p(): percentage.

© 2021 Marzuki AA et al. *JAMA Network Open*.

**eTable 10. Standard Regression Results for WCST Without Covariates (CTL vs MED- vs MED+)**

| Dependent Variable | Independent Variable | Estimate | p | BH-adjusted p | 95% CI | Test Used |
|---|---|---|---|---|---|---|
| Number of sets completed | Group | -0.23 | .40 | .56 | -0.77 – 0.31 | Linear Model |
| p(Perseverative errors) | Group | 0.04 | .54 | .56 | -0.09 – 0.17 | Binomial Model |
| p(Non-perseverative errors) | Group | 0.12 | .34 | .56 | -0.13 – 0.36 | Binomial Model |
| Mean RT | Group[a] | 138.29 | .002 | .02 | 50.40 – 226.18 | Linear Model |
| Failure to maintain set | Group | 0.29 | .14 | .33 | -0.10 - 0.69 | Linear Model |
| Number of trials needed to complete first set | Group | 1.13 | .56 | .56 | -2.70 – 4.95 | Linear Model |
| p(Unique errors) | Group[a] | 0.89 | .005 | .02 | 0.29 – 1.51 | Binomial Model |

Key: [a]significant at p<.05; MED-: Unmedicated OCD group; MED+: Medicated OCD group; M: mean; SD: standard deviation; df: degrees of freedom; BH: Benjamini-Hochberg Correction; CI: confidence interval; p(): proportion. Original p-values and BH corrected p-values are reported.

© 2021 Marzuki AA et al. *JAMA Network Open*.

## eTable 11. Standard Regression Results for WCST Covarying for Age, Gender, and IQ (CTL vs MED- vs MED+)

| Dependent Variable | Independent Variable | Estimate | p | BH-adjusted p | 95% CI | Test Used |
|---|---|---|---|---|---|---|
| Number of sets completed | Group<br>Gender<br>Age[a]<br>IQ[a] | -0.09<br>-0.62<br>0.62<br>0.03 | .65<br>.10<br>.006<br>.02 | .89<br>.49<br>.01<br>.04 | -0.51 – 0.32<br>-1.35 – 0.11<br>0.18 – 1.06<br>0.006 – 0.063 | Linear Model With Sandwich Estimator |
| p(Perseverative errors) | Group<br>Gender<br>Age[a]<br>IQ[a] | 0.006<br>0.08<br>-0.13<br>-0.01 | .92<br>.39<br>.003<br>.0004 | .92<br>.68<br>.01<br>.003 | -0.11 – 0.12<br>-0.10 – 0.25<br>-0.21 – -0.05<br>-0.02 – -0.006 | Binomial Model |
| p(Non-perseverative errors) | Group<br>Gender<br>Age[a]<br>IQ | 0.07<br>0.10<br>-0.21<br>-0.007 | .56<br>.60<br>.02<br>.36 | .89<br>.69<br>.02<br>.50 | -0.16 – 0.29<br>-0.27 – 0.46<br>-0.38 – -0.04<br>0.02 – 0.008 | Binomial Model |
| Mean RT | Group[a]<br>Gender<br>Age[a]<br>IQ[a] | 111.95<br>38.04<br>-137.03<br>-6.48 | .005<br>.54<br><.0001<br>.01 | .02<br>.69<br>.0001<br>.04 | 34.90 – 189.00<br>-84.02 – 160.10<br>-195.53 – -78.52<br>-11.36 – -1.59 | Linear Model |
| Failure to maintain set | Group<br>Gender<br>Age[a]<br>IQ | 0.24<br>0.32<br>-0.32<br>-0.02 | .22<br>.30<br>.04<br>.12 | .51<br>.68<br>.04<br>.22 | -0.15 – 0.62<br>-0.29 – 0.93<br>-0.61 – -0.02<br>-0.04 – 0.005 | Linear Model |
| Number of trials needed to complete first set | Group<br>Gender<br>Age[a]<br>IQ | 0.59<br>-1.20<br>-3.45<br>-0.05 | .76<br>.69<br>.02<br>.68 | .89<br>.69<br>.02<br>.69 | -3.24 – 4.42<br>-7.27 – 4.87<br>-6.36 – -0.54<br>-0.29 – 0.19 | Linear Model |
| p(Unique errors) | Group[a]<br>Gender<br>Age[a]<br>IQ | 0.96<br>-0.82<br>-0.68<br>0.008 | .003<br>.14<br>.004<br>.69 | .02<br>.49<br>.009<br>.69 | 0.36 – 1.60<br>-1.98 – 0.22<br>-1.14 – -0.24<br>-0.03 – 0.05 | Binomial Model |

Key: [a]significant at p<.05; MED-: Unmedicated OCD group; MED+: Medicated OCD group; M: mean; SD: standard deviation; df: degrees of freedom; BH: Benjamini-Hochberg Correction; CI: confidence interval; IQ: intelligence scores; p(): proportion. Original p-values and BH corrected p-values are reported.

© 2021 Marzuki AA et al. *JAMA Network Open*.

## eFigure 4. Group Comparisons (CTL vs MED- vs MED+) per WCST Measure

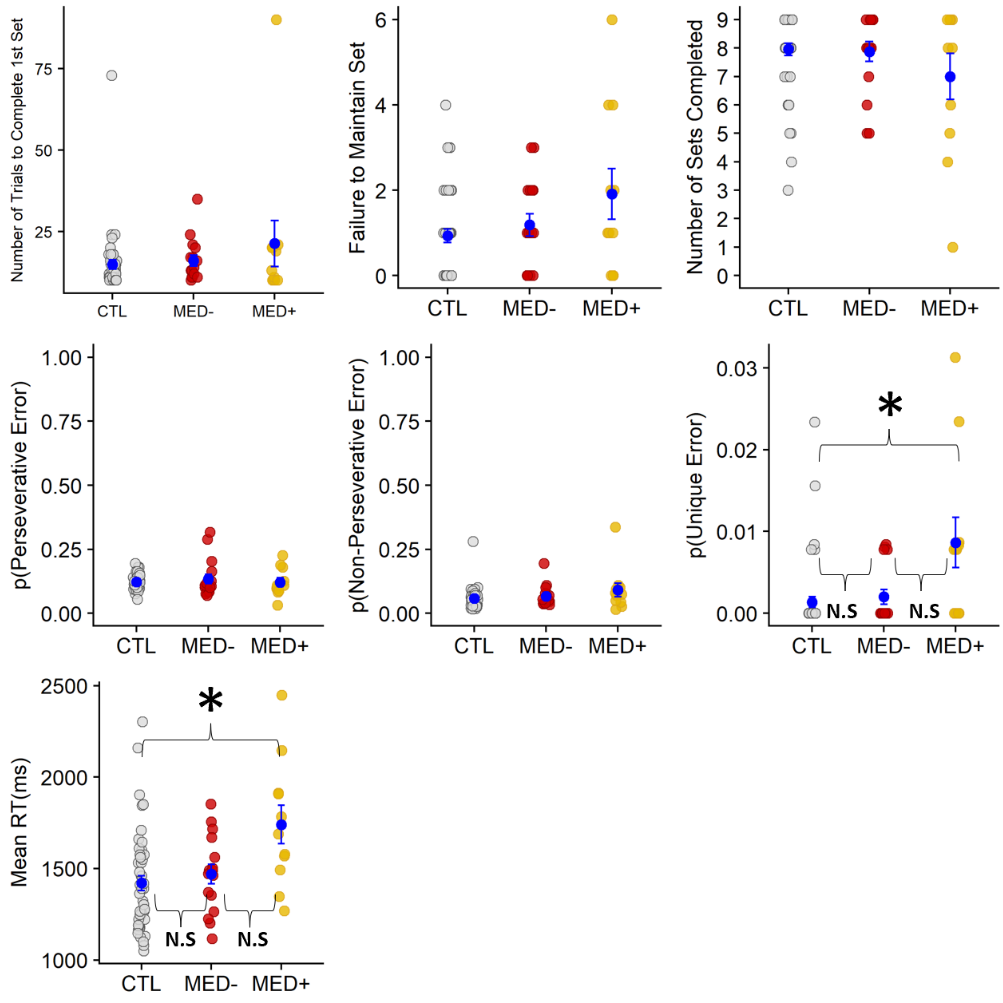


Key: N.S.: Non-significant; *p<.05, adjusted using Bonferroni correction. Error bars represent standard errors. Each point represents one subject.

© 2021 Marzuki AA et al. *JAMA Network Open*.

**Model Comparison**

Bridge sampling results to identify the best fitting reinforcement learning models for the PRL and WCST data are reported in eTables 12 and 13 respectively.

## eTable 12. Model Comparison Using Bridgesampling (PRL)

| Rank | Model | Parameters | Log Marginal Likelihood |
|---|---|---|---|
| 4 | 1 | $\alpha_{reinf}$, $\tau_{reinf}$ | -1966.29 |
| 2 | 2 | $\alpha_{rew}$, $\alpha_{pun}$, $\tau_{reinf}$ | -1925.94 |
| 1 | 3 | $\alpha_{rew}$, $\alpha_{pun}$, $\tau_{reinf}$, $\tau_{stim}$ | -1923.45 |
| 3 | 4 | $\alpha_{reinf}$, $\tau_{reinf}$, $\tau_{stim}$ | -1955.42 |
| 5 | 5 | ρ, φ, β | -1975.44 |

Notes: The log marginal likelihood obtained from bridge sampling is a comparison metric used to determine the best model. A numerically larger, i.e. less negative, log marginal likelihood is better. Model 3 was the best performing model here. Key – α: learning rate; rew: reward; pun: punishment; τstim: stimulus stickiness; τreinf: reinforcement sensitivity (inverse temperature); ρ: rho; φ: phi; β: beta (inverse temperature).

## eTable 13. Model Comparison Using Bridgesampling (WCST)

| **Rank** | **Model** | **Parameters** | **Log Marginal Likelihood** |
|---|---|---|---|
| 3 | 1 | RPDF | -206841.8 |
| 1 | 2 | RPD0 | -5952.683 |
| 4 | 3 | RP1F | -206924.8 |
| 2 | 4 | RRDF | -206711.6 |

Notes: The log marginal likelihood obtained from bridge sampling is a comparison metric used to determine the best model. A numerically larger, i.e. less negative, log marginal likelihood is better. Model 2 was the best performing model here. Key – R: reward rate; P: punishment rate; D: decision consistency; F: attentional focusing.

© 2021 Marzuki AA et al. *JAMA Network Open*.

**Parameter recovery for PRL**

Previously-fitted parameters used to generate the simulated data and their corresponding recovered values are presented in eTable 14 for OCD vs CTL, and in eTable 15 for MED- vs MED+ vs CTL. All generative parameter values fell strictly within their corresponding recovered 95% highest posterior density intervals (HDI), demonstrating that parameter values were successfully recovered. In addition, all 8 randomly initialised sampling chains were well-mixed, as indicated by the convergence diagnostic $\hat{R} \approx 1$ for every model parameter.

## eTable 14. Parameter Recovery Analysis With Simulated Data Generated by Best-Fit Computational Model (CTL vs OCD)

| Group | Parameter | Empirical Best-Fit | Simulated values | 95% HDI | R^ |
|---|---|---|---|---|---|
| CTL | Reward Rate | 0.28 | 0.31 | [0.27, 0.36] | 1.001 |
| | Punishment Rate | 0.58 | 0.59 | [0.56, 0.62] | 1 |
| | Reinforcement Sensitivity | 10.11 | 9.34 | [8.47, 10.27] | 1.001 |
| | Stimulus Stickiness | 0.41 | 0.44 | [0.33,0.55] | 1.002 |
| OCD | Reward Rate | 0.49 | 0.50 | [0.45, 0.54] | 1.001 |
| | Punishment Rate | 0.29 | 0.28 | [0.25, 0.31] | 1 |
| | Reinforcement Sensitivity | 5.20 | 5.24 | [4.93, 5.56] | 1.002 |
| | Stimulus Stickiness | 0.063 | 0.057 | [-0.046, 0.16] | 1.001 |

Key- CTL: control group, OCD: patient group, HDI: Highest density interval.

© 2021 Marzuki AA et al. *JAMA Network Open*.

**eTable 15. Parameter Recovery Analysis With Simulated Data Generated by Best-Fit Computational Model (CTL vs MED- vs MED+)**

| Group | Parameter | Empirical Best-Fit | Simulated values | 95% HDI | R^ |
|---|---|---|---|---|---|
| MED+ | Reward Rate | 0.5062 | 0.508 | [0.462, 0.553] | 1 |
| | Punishment Rate | 0.3014 | 0.302 | [0.273, 0.331] | 1.001 |
| | Reinforcement Sensitivity | 5.5741 | 5.516 | [5.186, 5.844] | 1.002 |
| | Stimulus Stickiness | 0.0139 | 0.031 | [0.079, 0.140] | 1.001 |
| MED- | Reward Rate | 0.473 | 0.496 | [0.453, 0.540] | 1 |
| | Punishment Rate | 0.26301 | 0.267 | [0.239, 0.299] | 1.001 |
| | Reinforcement Sensitivity | 4.694 | 4.719 | [4.437, 5.013] | 1.001 |
| | Stimulus Stickiness | 0.1279 | 0.084 | [0.012, 0.180] | 1 |
| CTL | Reward Rate | 0.2699 | 0.295 | [0.253, 0.341] | 1.002 |
| | Punishment Rate | 0.57905 | 0.592 | [0.560, 0.623] | 1 |
| | Reinforcement Sensitivity | 10.4279 | 9.615 | [8.709, 10.612] | 1.002 |
| | Stimulus Stickiness | 0.4121 | 0.453 | [0.338, 0.566] | 1.001 |

Key- CTL: control group, MED+: medicated OCD group, MED-: unmedicated OCD group, HDI: Highest density interval.

© 2021 Marzuki AA et al. *JAMA Network Open*.

**Correlations**

We conducted Pearson's correlations between demographic/clinical measures and WCST/PRL model parameter values to clarify whether model decision-making mechanisms are linked to clinical variables.

Within all subjects on the PRL (see eTable 16), anxiety, depression, and OCI scores showed significant associations with all parameter values IQ scores were also positively correlated with punishment rate and reinforcement sensitivity parameters. When considering only CTL, anxiety scores showed a negative association with punishment rate values and a positive association with stickiness values (see eTable 17). However, there were no significant correlations between all clinical measures and parameter values when considering only OCD (see eTable 18).

Within all subjects on the WCST, only age and IQ showed associations with the R and D model parameters (see eTable 19). When considering only CTL, OCI scores correlated with R parameter values, and negatively correlated with P and D parameter values (see eTable 20). When considering only OCD, OCI scores were positively correlated with P and D values, and CY-BOCS scores were positively correlated with P values (see eTable 21). However, we suspected that these correlations were confounded by age as OCD patients' OCI scores were positively correlated with age (r = 0.50; p = .01), and age also showed an association with P and D parameters (see eTable 21). Hence, we conducted further partial Pearson's correlations to control for the effects and age, which indeed rendered the relationships between OCD severity and model parameter values insignificant in the OCD group (all p >.05).

## eTable 16. Correlations Between Demographic/Clinical Measures and Model Parameters (PRL)—All Subjects

| | Age | IQ | Anxiety | Depression | OCI |
|---|---|---|---|---|---|
| **Reward Rate** | N.S | N.S | r = 0.58<br>*p* < .01 | r = 0.58<br>*p* < .01 | r = 0.64<br>*p* < .01 |
| **Punishment Rate** | N.S | r = 0.22<br>*p* = .03 | r = -0.39<br>*p* < .01 | r = -0.39<br>*p* < .01 | r = -0.49<br>*p* < .01 |
| **Reinforcement Sensitivity** | N.S | r = 0.24<br>*p* = .02 | r = -0.40<br>*p* < .01 | r = -0.40<br>*p* < .01 | r = -0.51<br>*p* < .01 |
| **Stickiness** | N.S | N.S | r = -0.58<br>*p* < .01 | r = -0.58<br>*p* < .01 | r = -0.70<br>*p* < .01 |

Key- N.S: Non-significant

## eTable 17. Correlations Between Demographic/Clinical Measures and Model Parameters (PRL)—CTL Only

| | Age | IQ | Anxiety | Depression | OCI |
|---|---|---|---|---|---|
| **Reward Rate** | r = 0.29<br>*p* = .03 | N.S | N.S | N.S | N.S |
| **Punishment Rate** | N.S | N.S | r = -0.28<br>*p* = .04 | N.S | N.S |
| **Reinforcement Sensitivity** | N.S | r = 0.29<br>*p* = .04 | N.S | N.S | N.S |
| **Stickiness** | N.S | N.S | r = 0.31<br>*p* = .02 | N.S | N.S |

Key- N.S: Non-significant

© 2021 Marzuki AA et al. *JAMA Network Open*.

## eTable 18. Correlations Between Demographic/Clinical Measures and Model Parameters (PRL)—OCD Only

| | **Age** | **IQ** | **Anxiety** | **Depression** | **OCI** | **CY-BOCS** |
|---|---|---|---|---|---|---|
| **Reward Rate** | N.S | N.S | N.S | N.S | N.S | N.S |
| **Punishment Rate** | N.S | r = 0.33 *p* = .02 | N.S | N.S | N.S | N.S |
| **Reinforcement Sensitivity** | N.S | N.S | N.S | N.S | N.S | N.S |
| **Stickiness** | N.S | r = 0.37 *p* < .01 | N.S | N.S | N.S | N.S |

Key- N.S: Non-significant

© 2021 Marzuki AA et al. *JAMA Network Open*.

**eFigure 5. Plots of Correlations Between Demographic/Clinical Measures and Model Parameters (PRL)**

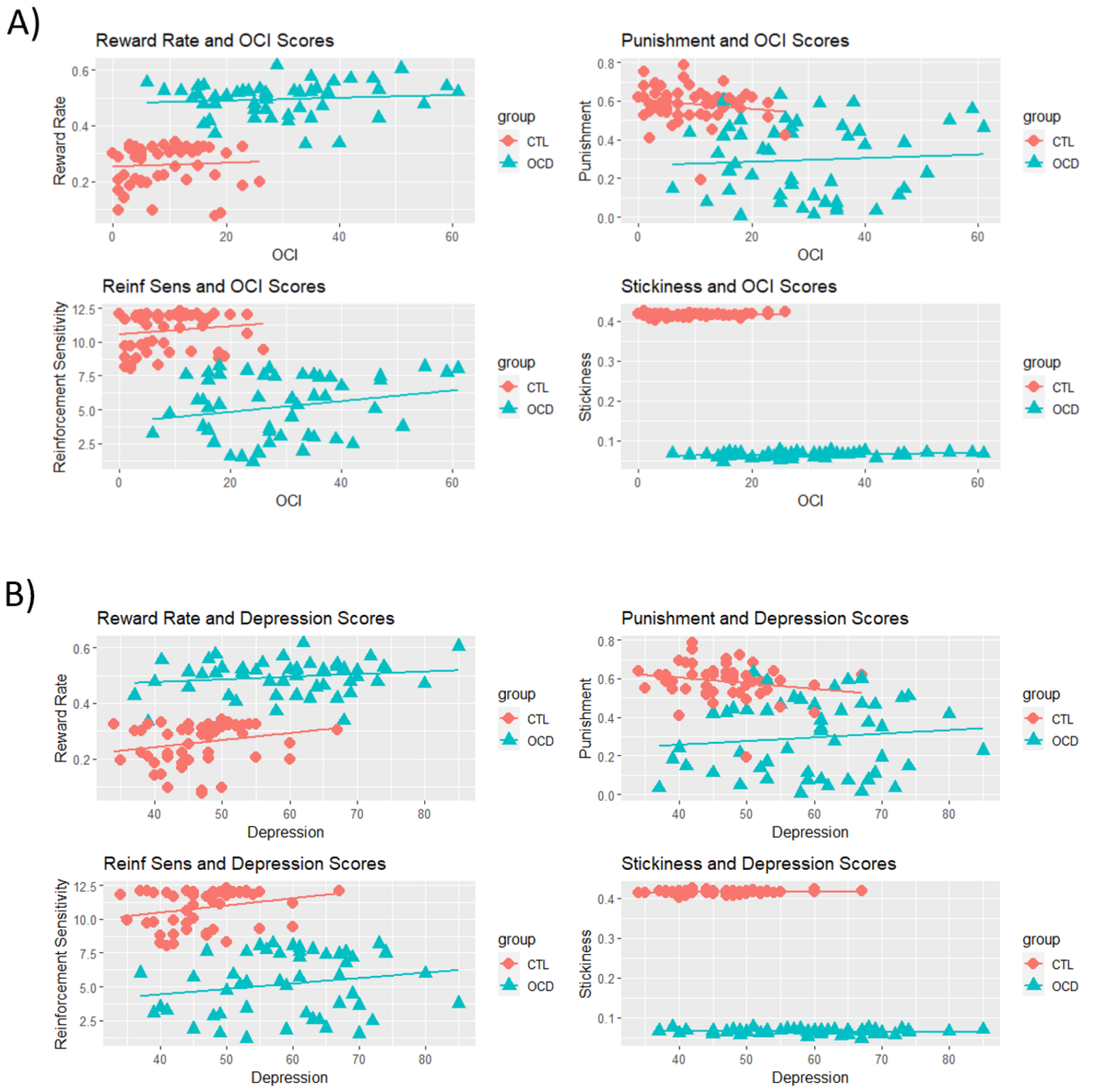


© 2021 Marzuki AA et al. *JAMA Network Open*.

C)

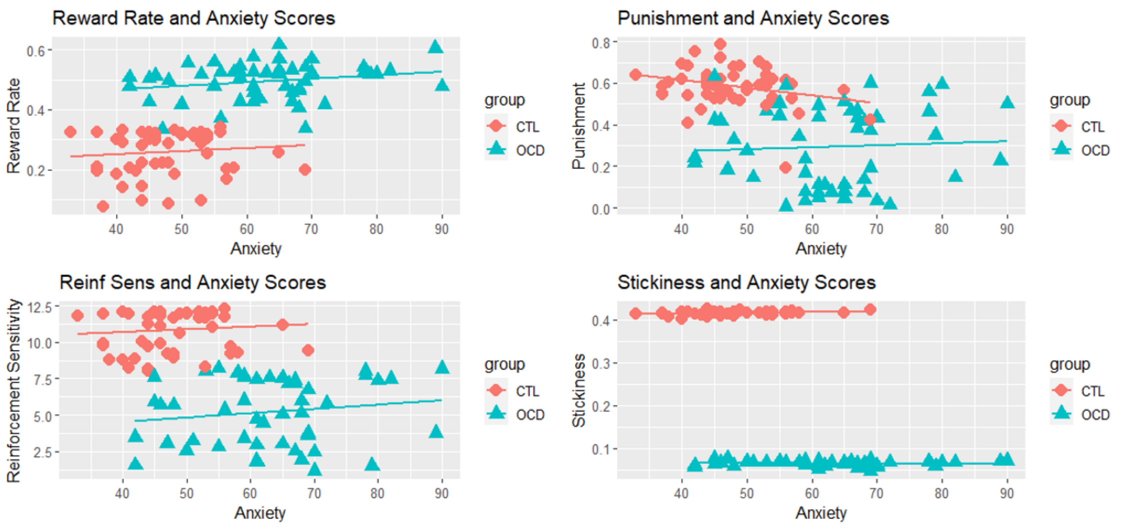


D)

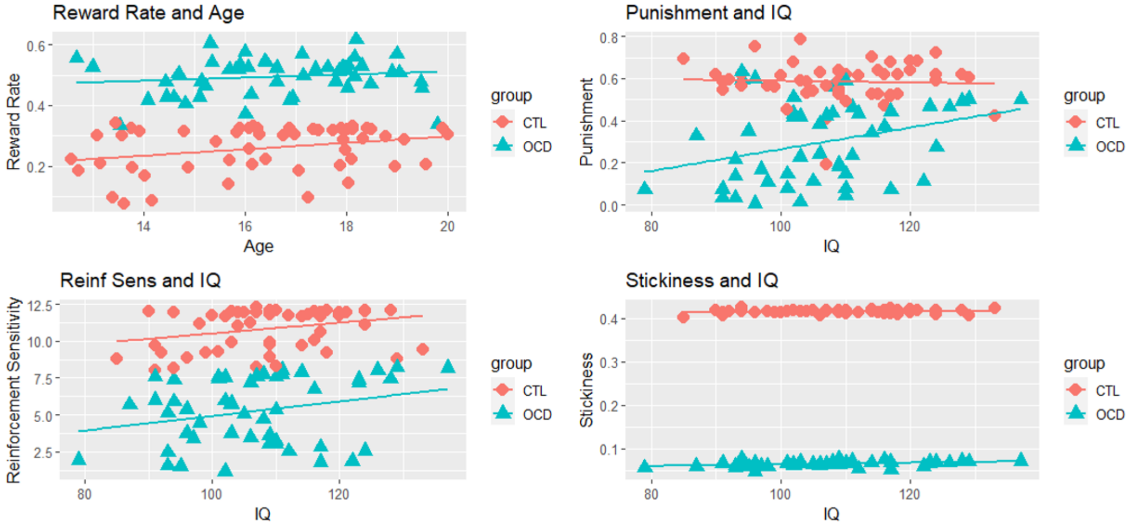


Plots of significant correlations for PRL task data. A) Correlations between OCI scores and model parameter values B) Correlations between depression scores (BDI) and model parameter values C) Correlations between anxiety scores (BAI) and model parameter values. D) Correlations between age/IQ and model parameter values.

© 2021 Marzuki AA et al. *JAMA Network Open*.

**eTable 19. Correlations Between Demographic/Clinical Measures and Model Parameters (WCST)—All Subjects**

| | **Age** | **IQ** | **Anxiety** | **Depression** | **OCI** |
|---|---|---|---|---|---|
| **R** | N.S | N.S | N.S | N.S | N.S |
| **P** | r = 0.39<br>$p < .01$ | r = 0.28<br>$p = .02$ | N.S | N.S | N.S |
| **D** | r = 0.40<br>$p < .01$ | r = 0.23<br>$p = .05$ | N.S | N.S | N.S |

Key- N.S: Non-significant

**eTable 20. Correlations Between Demographic/Clinical Measures and Model Parameters (WCST)—CTL Only**

| | **Age** | **IQ** | **Anxiety** | **Depression** | **OCI** |
|---|---|---|---|---|---|
| **R** | N.S | N.S | N.S | N.S | r = 0.33<br>$p = .02$ |
| **P** | N.S | N.S | N.S | N.S | r = -0.34<br>$p = .02$ |
| **D** | r = 0.34<br>$p = .02$ | N.S | N.S | N.S | r = -0.30<br>$p = .04$ |

Key- N.S: Non-significant

**eTable 21. Correlations Between Demographic/Clinical Measures and Model Parameters (WCST)—OCD Only**

| | **Age** | **IQ** | **Anxiety** | **Depression** | **OCI** | **CY-BOCS** |
|---|---|---|---|---|---|---|
| **R** | N.S | r = -0.50<br>$p = .02$ | N.S | N.S | N.S | N.S |
| **P** | r = 0.58<br>$p < .01$ | r = 0.52<br>$p = .01$ | N.S | N.S | r = 0.55<br>$p < .01$[a] | r = 0.55<br>$p < .01$[a] |
| **D** | r = 0.61<br>$p < .01$ | r = 0.47<br>$p = .03$ | N.S | N.S | r = 0.42<br>$p = .05$[a] | N.S |

Key- N.S: Non-significant. Note: [a]Not significant when controlling for age.

© 2021 Marzuki AA et al. *JAMA Network Open*.

**eFigure 6. Plots of Correlations Between Demographic/Clinical Measures and Model Parameters (WCST)**

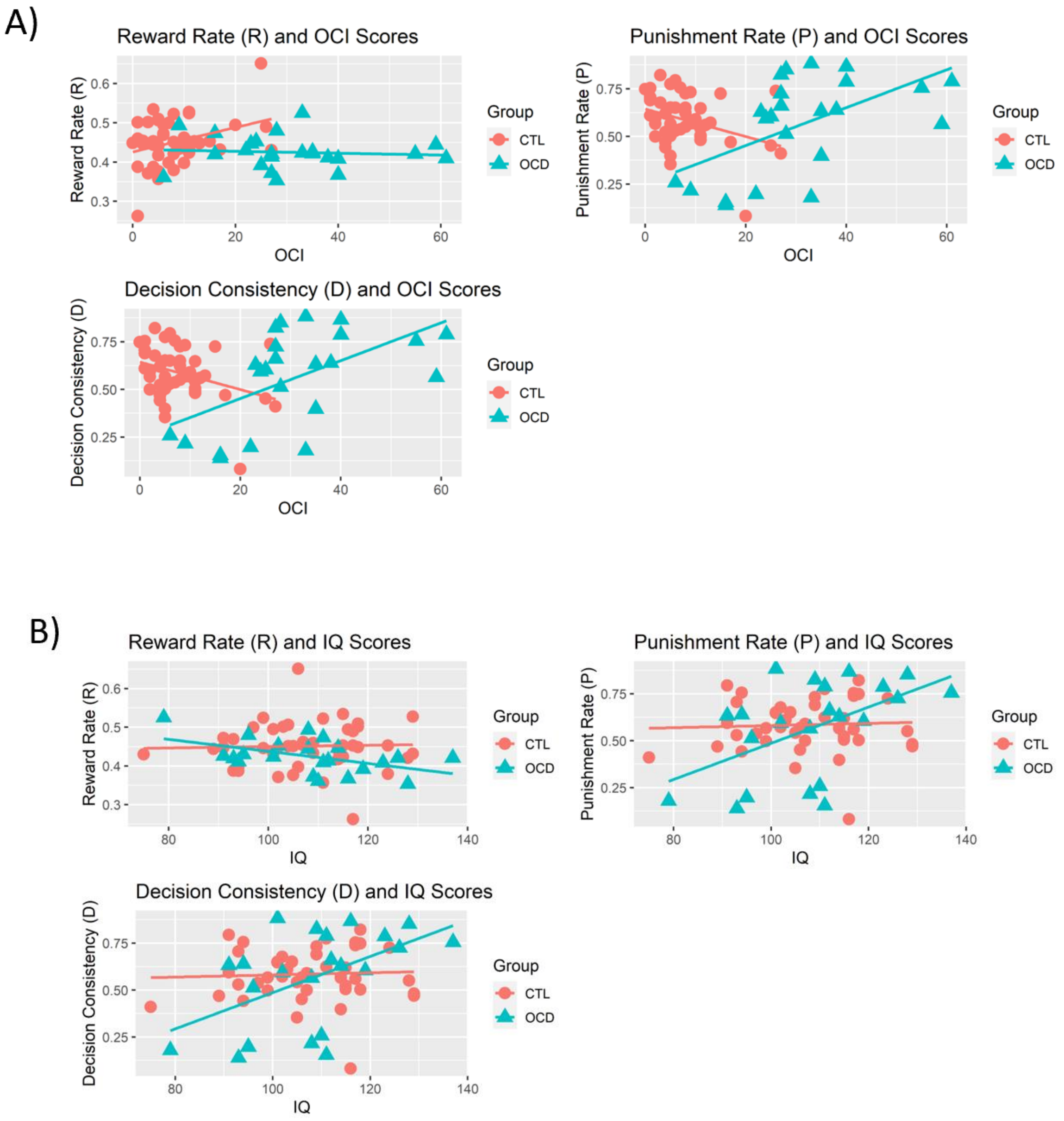


© 2021 Marzuki AA et al. *JAMA Network Open*.

C)

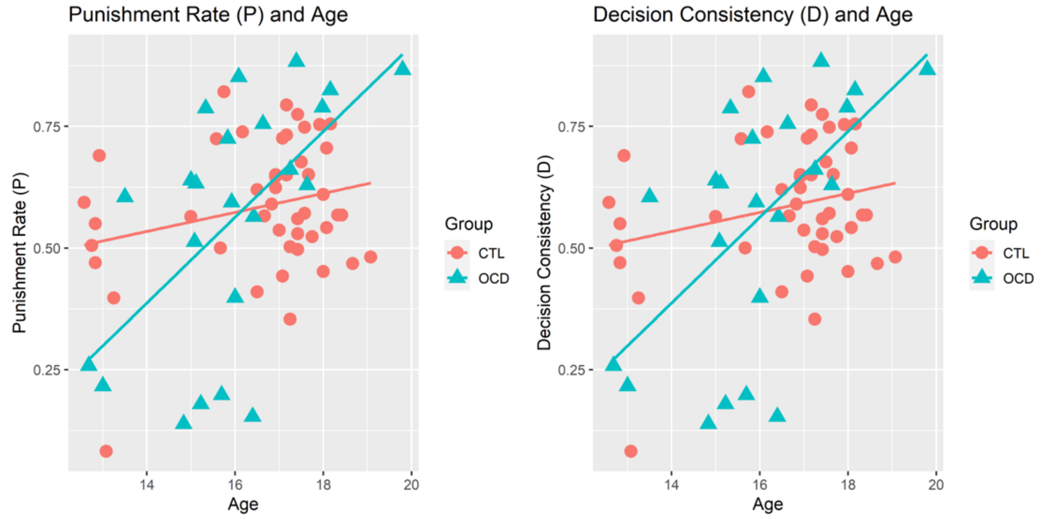


Plots of significant correlations for WCST data. A) Correlations between OCI scores and model parameter values B) Correlations between IQ and model parameter values C) Correlations between age and model parameter values.

© 2021 Marzuki AA et al. *JAMA Network Open*.

**Modelling Results For Participants that Completed Both Tasks**
We also modelled data obtained from 20 OCD and 17 CTL participants that completed both the WCST and PRL task. Results from these exploratory analyses revealed that OCD showed credibly higher reward rates (within a 90% HDI) and lower punishment rates (within a 95% HDI) than CTL on the PRL task, but the groups showed no differences on the reinforcement sensitivity and stickiness parameters. On the WCST, there were no differences between groups on all 3 parameters. See eFigure 5 for visualisation.

## eFigure 7. Modelling Results From 20 OCD and 17 CTL

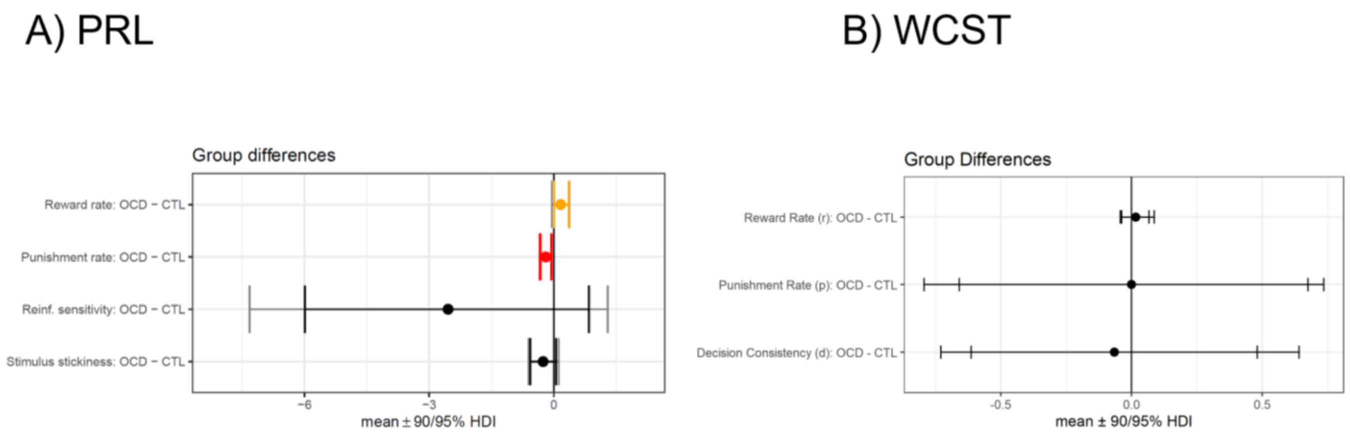


Note: Error bars in red indicate differences in posterior distributions between OCD and CTL are credible (not including 0) within a 95% HDI (highest density interval), while error bars in yellow indicate credible differences within a 90% HDI.

© 2021 Marzuki AA et al. *JAMA Network Open*.

**eReferences**


1. Sheehan, D. V. *et al.* The Mini-International Neuropsychiatric Interview (M.I.N.I.): The development and validation of a structured diagnostic psychiatric interview for DSM-IV and ICD-10. *J. Clin. Psychiatry* **59**, 22–33 (1998).
2. Sheehan, D. V. *et al.* Reliability and validity of the mini international neuropsychiatric interview for children and adolescents (MINI-KID). *J. Clin. Psychiatry* **71**, (2010).
3. Foa, E. B., Kozak, M. J., Salkovskis, P. M., Coles, M. E. & Amir, N. The validation of a new obsessive-compulsive disorder scale: The obsessive-compulsive inventory. *Psychol. Assess.* (1998) doi:10.1037/1040-3590.10.3.206.
4. Scahill, L. *et al.* Children's Yale-Brown Obsessive Compulsive Scale: Reliability and validity. *J. Am. Acad. Child Adolesc. Psychiatry* (1997) doi:10.1097/00004583-199706000-00023.
5. Beck, J., Beck, A., Jolly, J. & Steer, R. *Beck Youth Inventories – Second Edition for Children and Adolescents*. (NCS Pearson, Inc., 2005).
6. Wechsler, D. *Wechsler abbreviated scale of intelligence*. (TP Corporation, 1999).
7. Murphy, F., Smith, K., Cowen, P., Robbins, T. & Sahakian, B. The effects of tryptophan depletion on cognitive and affective processing in healthy volunteers. *Psychopharmacology (Berl).* **163**, 42–53 (2002).
8. Mueller, S. T. & Piper, B. J. The Psychology Experiment Building Language (PEBL) and PEBL Test Battery. *J. Neurosci. Methods* (2014) doi:10.1016/j.jneumeth.2013.10.024.
9. Bates, D., Maechler, M., Bolker, B. & Walker, S. lme4: Linear mixed-effects models using Eigen and S4. R package version 1.1-7, http://CRAN.R-project.org/package=lme4. *R Packag. version* (2014).
10. Andrés, S. *et al.* Changes in cognitive dysfunction in children and adolescents with obsessive-compulsive disorder after treatment. *J. Psychiatr. Res.* (2008) doi:10.1016/j.jpsychires.2007.04.004.
11. Geller, D. A. *et al.* Neurocognitive function in paediatric obsessive-compulsive disorder. *World J. Biol. Psychiatry* (2018) doi:10.1080/15622975.2017.1282173.
12. Gläscher, J., Adolphs, R. & Tranel, D. Model-based lesion mapping of cognitive control using the Wisconsin Card Sorting Test. *Nat. Commun.* **10**, (2019).
13. Isik Taner, Y., Erdogan Bakar, E. & Oner, O. Impaired executive functions in paediatric obsessive-compulsive disorder patients. *Acta Neuropsychiatr.* (2011) doi:10.1111/j.1601-5215.2011.00562.x.
14. Somsen, R. J. M. The development of attention regulation in the Wisconsin Card Sorting Task: PAPER. *Dev. Sci.* **10**, 664–680 (2007).
15. Somsen, R. J. M., Van Der Molen, M. W., Jennings, J. R. & Van Beek, B. Wisconsin Card Sorting in adolescents: Analysis of performance, response times and heart rate. *Acta Psychol. (Amst).* **104**, 227–257 (2000).
16. Zeileis, A. Econometric computing with HC and HAC covariance matrix estimators. *J. Stat. Softw.* (2004) doi:10.18637/jss.v011.i10.
17. Lüdecke, D., Ben-Shachar, M., Patil, I., Waggoner, P. & Makowski, D. performance: An R Package for Assessment, Comparison and Testing of Statistical Models. *J. Open Source Softw.* **6**, 3139 (2021).
18. Sutton, R. S. & Barto, A. G. *Reinforcement learning: An Introduction*. *MIT Press* (2018).
19. Clarke, H. F. *et al.* Orbitofrontal dopamine depletion upregulates caudate dopamine and alters behavior via changes in reinforcement sensitivity. *J. Neurosci.* **34**, 7663–7676 (2014).
20. Hauser, T. U. *et al.* Increased fronto-striatal reward prediction errors moderate decision making in obsessive-compulsive disorder. *Psychol. Med.* **47**, 1246–1258 (2017).
21. Hauser, T. U., Iannaccone, R., Walitza, S., Brandeis, D. & Brem, S. Cognitive flexibility in

adolescence: Neural and behavioral mechanisms of reward prediction error processing in adaptive decision making during development. *Neuroimage* **104**, 347–354 (2015).

22. Eckstein, M., Master, S., Dahl, R., Wilbrecht, L. & Collins, A. The Unique Advantage of Adolescents in Probabilistic Reversal: Reinforcement Learning and Bayesian Inference Provide Adequate and Complementary Models. *bioRxiv* 2020.07.04.187971 (2020).

23. Kanen, J. W., Ersche, K. D., Fineberg, N. A., Robbins, T. W. & Cardinal, R. N. Computational modelling reveals contrasting effects on reinforcement learning and cognitive flexibility in stimulant use disorder and obsessive-compulsive disorder: remediating effects of dopaminergic D2/3 receptor agents. *Psychopharmacology (Berl).* 2337–2358 (2019) doi:10.1007/s00213-019-05325-w.

24. Pessiglione, M. & Delgado, M. R. The good, the bad and the brain: Neural correlates of appetitive and aversive values underlying decision making. *Curr. Opin. Behav. Sci.* **5**, 78–84 (2015).

25. Den Ouden, H. E. M. *et al.* Dissociable Effects of Dopamine and Serotonin on Reversal Learning. *Neuron* **80**, 1090–1100 (2013).

26. Wilson, R. C. & Collins, A. G. E. Ten simple rules for the computational modeling of behavioral data. *Elife* **8**, 1–33 (2019).

27. Bishara, A. J. *et al.* Sequential learning models for the Wisconsin card sort task: Assessing processes in substance dependent individuals. *J. Math. Psychol.* (2010) doi:10.1016/j.jmp.2008.10.002.

28. Farreny, A. *et al.* Study of positive and negative feedback sensitivity in psychosis using the Wisconsin Card Sorting Test. *Compr. Psychiatry* **68**, 119–128 (2016).

29. Plummer, M. JAGS : A Program for Analysis of Bayesian Graphical Models. in *Proceedings of the 3rd International Workshop on Distributed Statistical Computing (DSC 2003)* (2003).

30. Gronau, Q. F. *et al.* A tutorial on bridge sampling. *J. Math. Psychol.* (2017) doi:10.1016/j.jmp.2017.09.005.

31. Gelman, A., Hill, J. & Yajima, M. Why We (Usually) Don't Have to Worry About Multiple Comparisons. *J. Res. Educ. Eff.* (2012) doi:10.1080/19345747.2011.618213.

© 2021 Marzuki AA et al. *JAMA Network Open*.
